# Assessment of Intracellular Delivery Potential of Novel Sustainable Poly(δ-decalactone)-Based Micelles

**DOI:** 10.3390/pharmaceutics12080726

**Published:** 2020-08-02

**Authors:** Kuldeep Kumar Bansal, Ezgi Özliseli, Gaurav Kumar Saraogi, Jessica M. Rosenholm

**Affiliations:** 1 Pharmaceutical Sciences Laboratory, Faculty of Science and Engineering, Åbo Akademi University, 20520 Turku, Finland; Ezgi.Ozliseli@abo.fi (E.Ö.); jessica.rosenholm@abo.fi (J.M.R.); 2 School of Pharmacy, University of Nottingham, University Park, Nottingham NG7 2RD, UK; 3 NMIMS School of Pharmacy and Technology Management, Shirpur (Maharashtra) 425405, India; gauravsaraogi13@gmail.com

**Keywords:** poly(δ-decalactone), functionalised micelles, targeted drug delivery, renewable polymer, caveolae-mediated endocytosis, intracellular delivery

## Abstract

Biodegradable polymers from renewable resources have attracted much attention in recent years within the biomedical field. Lately, poly(δ-decalactone) based copolymer micelles have emerged as a potential drug delivery carrier material as a sustainable alternative to fossil-based polymers. However, their intracellular drug delivery potential is not yet investigated and therefore, in this work, we report on the synthesis and cellular uptake efficiency of poly(δ-decalactone) based micelles with or without a targeting ligand. Folic acid was chosen as a model targeting ligand and Rhodamine B as a fluorescent tracer to demonstrate the straightforward functionalisation aspect of copolymers. The synthesis of block copolymers was accomplished by a combination of facile ring-opening polymerisation and click chemistry to retain the structure uniformity. The presence of folic acid on the surface of micelles with diameter ~150 nm upsurge the uptake efficiency by 1.6 fold on folate receptor overexpressing MDA-MB-231 cells indicating the attainment of targeting using ligand functionality. The drug delivery capability of these carriers was ascertained by using docetaxel as a model drug, whereby the in vitro cytotoxicity of the drug was significantly increased after incorporation in micelles 48 h post incubation. We have also investigated the possible endocytosis route of non-targeted micelles and found that caveolae-mediated endocytosis was the preferred route of uptake. This work strengthens the prospect of using novel bio-based poly(δ-decalactone) micelles as efficient multifunctional drug delivery nanocarriers towards medical applications.

## 1. Introduction

The majority of pharmacologically active compounds are known to act on targets located within the cell for the treatment of diseases. Intracellular drug targeting strategies include targeting the cytoplasm, endosomes, mitochondria, lysosomes, nucleus, and so forth [1]. Consequently, several nanocarriers have been utilised to deliver drugs intracellularly to attain an enhanced therapeutic response compared to drug alone. Ligand-mediated targeting, also known as active targeting is the most common approach used to deliver drugs intracellularly via nanocarriers [2]. In cancer therapies, superior antitumor activities of drug-loaded nanocarriers with active targeting capabilities have been reported due to their enhanced cellular internalisation via receptor-mediated endocytosis. The most common ligands used for targeted drug delivery are sugars, antibodies, nucleic acids, proteins, peptides, and small molecules such as vitamins [3]. Folic acid (FA) as a targeting ligand is the most studied molecule utilised for active targeting in targeted therapy to deliver drug-loaded nanocarriers at the site of action. Several FA-conjugated drug delivery carriers such as liposomes, nanoparticles, micelles, dendrimers, and carbon nanotubes have been investigated for targeted cancer therapy [4]. Based on the excellent in vitro and in vivo results, a few folic acid-based drug delivery systems have been entered in clinical trials [5].

Although liposomes, nanoparticles, and other drug delivery systems have been successfully used for targeted therapy, the use of polymeric micelles to deliver cytotoxic drugs have their own advantages [6]. Similarly to other nanoscaled drug delivery systems, polymeric micelles have the ability to passively target the tumour via the enhanced permeability and retention (EPR) effect, as well as provide the opportunity of active targeting through the coupling of ligands. After tumour accumulation, polymeric micelles can selectively deliver the drugs to their subcellular targets by acting as intracellular “Trojan horses” and thus, could solve the drug resistance problem [7]. Additionally, micelles have been shown to circumvent recognition by the drug efflux pump (e.g., P-glycoprotein) and can consequently overcome multidrug resistance in cancer cells, which, in turn, can enhance the intracellular concentration of drugs [8].

Folic acid-decorated polymeric micelles have been widely studied as a potential targeted therapy for cancer treatment due to the upregulation of folate receptors (FR) in many types of human cancers and especially in triple-negative breast cancer (TNBC). TNBC lack in prevalent targeted receptors including the oestrogen receptor and the progesterone receptor and in the amplification of the HER-2/Neu receptor, albeit expressing high amounts of folic acid receptors [9]. Due to the functional group chemistry of FA, its conjugation with end-functionalised block copolymers is facile. Numerous reports have to date been published proposing the effective treatment of cancer via folate conjugated polymeric micelles [10].

Polymeric micelles are usually fabricated using amphiphilic block copolymers. Poly (ethylene glycol) (PEG) is typically the polymer of choice to be used as a hydrophilic block, whereas the hydrophobic block can be chosen based on the required application. In recent years, biodegradable hydrophobic polymers synthesised from renewable feedstock have gained considerable interest among researchers as a sustainable alternative, considering the depletion of fossil fuels and environmental pollution and thus, these can have both ecological and economic benefits [11]. Consequently, we have recently reported the synthesis of biodegradable block copolymers based on renewable δ-decalactone via an environmentally friendly route and investigated its drug delivery capability. These polymers have successfully enhanced the aqueous solubility of hydrophobic drugs, and demonstrate low toxicity in vitro and in vivo within working concentration range [12,13,14]. In addition to that, we have also explored the drug delivery capability of δ-decalactone homopolymer (an amorphous polymer) by utilising it as an oil to fabricate nanoemulsion [15]. Due to the higher hydrophobicity of poly(δ-decalactone) (PDL)-based amphiphilic copolymers, lower critical micelle concentration, higher drug loading and a faster and complete release of hydrophobic guests are expected from them compared to their counterparts of similar molecular weight [12,13,16]. Moreover, due to the amorphous nature of PDL, a higher degradation rate in aqueous medium is expected compared to semicrystalline and crystalline poly(esters), which will be beneficial in certain drug delivery applications [13,17]. Thus, considering their promising capabilities as a drug delivery carrier, investigation of their ligand-mediated targeting ability to cancer cells has been proposed.

Therefore, in this study, three different block copolymers, i.e., FA-tagged block copolymer poly(ethylene glycol)-b-poly(δ-decalactone) (FA-PEG-b-PDL) for ligand-mediated targeting, rhodamine (RhB) tagged copolymer (RhB-PEG-b-PDL) for tracking, and non-functionalised PEG-b-PDL as a control formulation have been synthesised via facile chemistry. Mixed micelles of the functionalised and non-functionalised PEG-b-PDL copolymers with RhB-PEG-b-PDL were prepared (abbreviated as PDL-FA and PDL) and evaluated for their folate-mediated targeting efficiency on MDA-MB-231 TNBC cell line in vitro. Docetaxel (DTX) was used as a model drug to evaluate the drug delivery capability of fabricated nanocarriers. To the best of our knowledge, this is the first report demonstrating the cellular uptake behaviour of block copolymer micelles of amorphous PDL. This study could pave the way of utilizing PDL based nanocarriers for diverse intracellular drug delivery applications.

## 2. Materials and Methods

### 2.1. Materials

Poly(ethylene glycol) methyl ether (mPEG, Mn = 5.0 KDa), δ-decalactone (≥98%), 1,5,7-triazabicyclo [4.4.0]dec-5-ene (TBD) (98%), propargyl alcohol (99%), anhydrous pyridine (99.8%), p-toluenesulfonyl chloride (≥99%), sodium azide (≥99.5%), folic acid (≥97%), rhodamine B isothiocyanate(mixed isomer), N,N′-dicyclohexylcarbodiimide (99%), N-hydroxysuccinimide (98%), trimethylamine (≥99%), copper (I) bromide (99.9%), and all the solvents were purchased from Sigma Aldrich. DTX was generously provided as a gift sample by Zydus Cadilla, Ahmedabad, India. N_3_-PEG-NH_2_.TFA salt was purchased from JenKemUSA, which has ≥95% of amine substitution and >90% of azide substitution.

#### 2.1.1. Instruments

##### Nuclear Magnetic Resonance (NMR) Spectroscopy

NMR spectra were recorded at 400 MHz using a Bruker NMR spectrometer in deuterated chloroform (99.8% D) and dimethyl sulfoxide (99.9% D). Spectra were assessed using MestReNova 6.0.2 (Mestrelab Research S.L.) [18].

##### Size Exclusion Chromatography (SEC)

Size exclusion chromatography was carried out using a Polymer Laboratories GPC 50 instrument fitted with a differential refractive index detector. The number-average molar mass (M_n_), weight average molar mass (M_w_) and polydispersity (Đ, M_w_/M_n_) were measured by SEC using HPLC-grade chloroform as eluent at 30 °C with 1 mL min^−1^ flow rate. PLgel guard column (50 × 7.5 mm, 8 μm) followed by a pair of PLgel Mixed-D columns (300 × 7.5 mm, 8 μm) were used for separation of the sample. Polystyrene standards of known M_n_ and Đ in the range of 100 Da– 500 kDa were used to calibrate the column. Molar mass and polydispersity were calculated using Polymer Labs Cirrus 3.0 software [18].

##### MALDI-TOF Mass Spectrometry (MALDI-TOF Mass)

Matrix-assisted laser desorption/ionisation time-of-flight (MALDI-TOF) mass spectroscopy was performed on an Ultraflex III MALDI-TOF spectrometer with N_2_ laser of 337 nm and pulses of 3 ns. Sodium trifluoroacetate was used as a dopant, and the matrix used in the experiment was trans-2-[3-(4-tert-Butylphenyl)-2-methyl-2-propenylidene] malononitrile (DCTB). Samples were prepared by mixing 5mg/mL of sample in 10mg/mL of the matrix in chloroform [18].

### 2.2. Methods

#### 2.2.1. Synthesis of Azide Terminated Poly(ethylene glycol) Methyl Ether (mPEG-N_3_)

Synthesis of methoxy-PEG-N_3_ using poly(ethylene glycol) methyl ether was accomplished in two steps via a reported procedure [19]. Briefly, p-toluene sulfonyl chloride (0.76 g, 4.00 mmol) was added under an inert atmosphere to the solution of mPEG (2.00 g, 0.40 mmol) in pyridine (10.0 mL), and stirred for 24 h at RT. The mixture was then precipitated four times in cold diethyl ether and dried under vacuum to obtain a white, solid product, i.e., tosylated mPEG (mPEG-OTs) (1.64 g, 82%). Next, a solution of mPEG-OTs (1.50 g, 0.25 mmol) was prepared in DMSO (10.0 mL), and sodium azide (203.50 mg, 3.13 mmol) was added to it. The mixture was then stirred for 24 h at RT under an inert atmosphere. For purification, the reaction mixture was dissolved in dichloromethane (20.0 mL) and the organic layer was washed with cold distilled water (50.0 mL × 3), followed by cold 6 M hydrochloric acid solution (50.0 mL × 2) and then again with cold distilled water (50.0 mL × 2). The organic layer was separated and dried with anhydrous MgSO_4,_ followed by filtration, and the solvent was evaporated in vacuum. The obtained residue was further precipitated in cold diethyl ether, and remaining solvent traces were removed by drying in a vacuum oven. An off-white solid product (1.2 g, 78%) was obtained, and the conversion of tosyl to azide product as calculated by ^1^HNMR was 89%.

mPEG-OTs-^1^H NMR (400 MHz, CDCl_3_) δ (ppm) 7.83 (Aromatic–CH, d, 2H), 7.35 (Aromatic–CH, d, 2H), 4.23–4.13 (CH_2_–CH_2_–O-Tosyl,t, 2H), 3.66 (O–CH_2_–CH_2_–O, s, 508H), 3.40 (O–CH_3_, s, 3H), 2.47 (Aromatic–CH_3_, s, 3H).

mPEG-N_3_-^1^H NMR (400 MHz, CDCl_3_) δ (ppm) 3.67 (O–CH_2_–CH_2_–O, s,508H), 3.43–3.36 (O–CH_3_, CH_2_–CH_2_–N_3_, m, 5H)

#### 2.2.2. Synthesis of Folate Conjugated Poly(Ethylene Glycol) (FA-PEG-N_3_)

Conjugation of folic acid on to the N_3_-PEG-NH_2_.TFA salt was performed in a single step by following a reported method [20]. Briefly, a solution of folic acid (0.055 g, 0.12 mmol) was prepared in anhydrous DMSO (2.0 mL) in the absence of light. Triethylamine (0.6 mL), *N,N*′-dicyclohexylcarbodiimide (DCC) (0.03 g, 0.15 mmol) and *N*-hydroxysuccinimide (NHS) (0.02 g, 0.15 mmol) were then added to the folic acid solution and stirred overnight at room temperature (RT) in the dark under inert atmosphere. Separately, N_3_-PEG-NH_2_.TFA salt (0.25 g, 0.05 mmol) was dissolved in anhydrous DMSO (2.0 mL) contained triethylamine (0.1 mL) and stirred for 2 h to activate N_3_-PEG-NH_2_. PEG solution was then added to the solution of N-hydroxysuccinimidyl-ester of folic acid and stirred for 24 h at RT in the absence of light. The obtained solution was precipitated several times in cold diethyl ether, and the solvent residue was removed under vacuum. The recovered dry yellow solid was then dissolved in DCM (5.0 mL, a nonsolvent for folic acid) and centrifuge to remove the free folic acid. The supernatant was collected, and the solvent was removed under vacuum. The obtained product was then dissolved in HPLC-grade water (5.0 mL), and pH was adjusted to 3.0 (approx.) using hydrochloric acid (1 M) to remove any available free folic acid via precipitation. The solution was then filtered with 0.22 µm syringe filter and dialysed (MWCO of dialysis bag—1000 Da) against PBS (pH-7.4) for three days to purify the product and then for two days against deionised (DI) water to remove salts. The final solution was then filtered and freeze-dried to obtain the folate conjugate PEG-N_3,_ which was light yellow (251 mg, 92%). The amount of conjugated folic acid in the final product, i.e., folate–PEG_5000_-N_3_ (FA-PEG-N_3_) was determined using a UV-Vis spectrophotometer, and the concentration of folic acid was calculated using a preprepared standard calibration curve of folic acid in PBS at λ_max_ of 280 nm.

FA-PEG-N_3_ – ^1^H NMR (400 MHz, DMSO-d_6_ contained a few drops of D_2_O) δ (ppm) 8.65 (Ar–N–CH, d, 0.8H), 7.63 (Ar–CH, d, 1.7H), 6.64 (Ar–CH, d, 1.7H), 4.50 (NH–CH_2_, d, 1.7H), 4.40–4.19 (CH_2_–CH–COOH, m, 0.8H), 3.49 (O–CH_2_–CH_2_–O, s, 498H),3.34–3.26 (CH_2_–CH_2_–N_3_, m, 2H), 3.25–3.10 (CH_2_–CH_2_–NH–COO, m, 1.6H), 2.43–2.10 (CH_2_–CH_2_–CH–COOH, m, 1.6H), 2.10–1.75 (CH_2_–CH_2_–CH–COOH, m, 2.0H).

MALDI-TOF MASS: N_3_-PEG-NH_2_.TFA-*m/z.* Calculated—5000, Found—4706 [M]^+^.

FA-PEG-N_3_– *m/z.* Calculated—5129, Found—5176 [M + 2Na]^+^.

#### 2.2.3. Synthesis of Rhodamine B Conjugated Poly (ethylene glycol) (RhB-PEG-N_3_)

Conjugation of Rhodamine B *iso*thiocyanate (RhB) on PEG was done via a reported method [21]. Briefly, N_3_-PEG-NH_2_.TFA salt (0.25 g, 0.05 mmol) was dissolved in anhydrous DMSO (2.0 mL) containing triethylamine (0.1 mL) and stirred for 2 h to activate it. Rhodamine B *iso*thiocyanate (0.053 g, 0.10 mmol) was then added to the above solution, stirred for 24 h before precipitation in cold diethyl ether (×4). The solvent residue was then evaporated under vacuum, and the obtained red solid was dissolved in HPLC grade water (5.0 mL) and dialysed (MWCO of dialysis bag—1000 Da) for six days to remove unconjugated rhodamine B *iso*thiocyanate. The obtained solution was then freeze-dried to yield rhodamine-conjugated PEG-N_3_ (RhB–PEG_5000_-N_3_, 231 mg, 84%). To determine the concentration of rhodamine B in conjugate, RhB–PEG_5000_-N_3_ was analysed on UV-Vis spectrophotometer, and the amount of rhodamine B was calculated using a preprepared standard calibration curve of rhodamine B at λ_max_ of 552 nm.

^1^HNMR (400 MHz, CDCl_3_) δ (ppm) 6.76–6.25 (Ar–CH, m, 5.3H), 3.66 (O–CH_2_–CH_2_–O, s, 509H), 3.45–3.35 (CH_2_–CH_2_–N_3_, m, 2H), 1.44–1.01 (–N–CH_2_–CH_3_, m, 11.4H).

^1^HNMR (400 MHz, DMSO-d_6_) δ (ppm) 10.57 (Ar–COOH, s, 0.5 H), 8.01 (Ar–CH, m, 2.7H), 6.87 (Ar–CH, m, 5.4H), 3.52 (O–CH_2_–CH_2_–O, s, 500H), 1.14 (–N–CH_2_–CH_3_, d, 11H).

MALDI-TOF MASS: RhB-PEG-N_3_ – *m*/*z.* Calculated—5243, Found—5361 [M +TFA].

#### 2.2.4. Synthesis of Propargyl-PDL

The synthesis and characterisation of Propargyl PDL was already reported in one of our previous publications, and the same polymer was used in this study [13]. The degree of polymerisation selected for the synthesis of propargyl-PDL was 100. The *M_n_* obtained by SEC was used for further calculations.

#### 2.2.5. Synthesis of Block Copolymers via Click Chemistry

The block copolymers using hydrophilic PEG and hydrophobic PDL blocks were synthesised via azide-alkyne click chemistry using copper as a catalyst [22]. Briefly, propargyl-PDL (1.82 g, 0.24 mmol), mPEG-N_3_ (0.48 g, 0.10 mmol) and 2,2′-Bipyridyl (0.02g, 0.10 mmol) were dissolved in 2.0 mL of dimethylacetamide (DMAc) under nitrogen atmosphere. Copper (I) bromide (0.7 mg, 0.005 mmol) was then added to the above solution followed by addition of a saturated solution of sodium ascorbate (10 µL) in water (which was diluted to 100 µL with DMAc) and stirred for 48 h. The obtained reaction mixture was then precipitated four times in petroleum ether to remove unconjugated propargyl-PDL and 2,2′-bipyridyl. The obtained precipitate was then dissolved in a minimum quantity of CHCl_3_and centrifuged (15000 rpm, 2 min.) to remove copper and sodium ascorbate. The supernatant was collected, and the solvent was evaporated in vacuum to yield the block copolymer mPEG-b-PDL, which was a hard wax-like material (1.0 g, 87%). A similar procedure was followed to synthesise FA-PEG-b-PDL and RhB-PEG-b-PDL using FA-PEG-N_3_ (200 mg) and RhB-PEG-N_3_ (180 mg) respectively. The quantity of 2,2′-bipyridyl (1 equivalent) and copper (I) bromide (0.05 equivalent) was calculated based on the PEG concentration. The percentage yield observed for FA-PEG-b-PDL was 81% (398 mg) while 79% (361 mg) yield was observed for RhB-PEG-b-PDL.

mPEG-b-PDL:

^1^H NMR (400 MHz, CDCl_3_) δ (ppm) 7.80 (Triazole-CH, s, 1H), 5.23 (COO–CH_2_–Triazole, s,2H), 5.02–4.78 (CH–O–CO, m, 42H), 4.66–4.46 (CH_2_–CH_2_–triazole, t, 2H), 3.93–3.86 (CH_2_–CH_2_–triazole, t, 2H), 3.66 (O–CH_2_–CH_2_–O,s, 507H), 3.39 (O–CH_3_,s, 3H), 2.42–2.21 (O–CO–CH_2_,m, 85H), 1.77–1.40 (CH_2_–CH_2_–CH–CH_2_, m, 254H), 1.40–1.16 (CH_2_–CH_2_–CH_2_–CH_3_,m, 259H), 0.89 (CH_3_, t, 129H).

FA–PEG–b–PDL:

^1^HNMR (400 MHz, CDCl_3_) δ (ppm) 7.80 (Triazole–CH, s, 1H), 5.22 (COO–CH_2_–Triazole, s, 2H), 5.00–4.80 (CH–O–CO, m,42H), 4.65–4.49 (CH_2_–CH_2_–triazole, t,2H), 3.96–3.87 (CH_2_–CH_2_–triazole,t,2H), 3.65 (O–CH_2_–CH_2_–O,s, 500H), 2.48–2.19 (O–CO–CH_2_,m, 86H), 1.89–1.40 (CH_2_–CH_2_–CH–CH_2_, m, 253H), 1.40–1.14 (CH_2_–CH_2_–CH_2_–CH_3_,m, 259H), 1.03–0.76 (CH_3_,t, 131H).

^1^HNMR (400 MHz, DMSO–d_6_) δ (ppm) 8.66 (Ar–N–CH, s, 1H), 8.06 (Triazole–CH, s, 1H), 7.66 (Ar–CH, s, 1.7H), 6.66 (Ar–CH,s, 2H), 4.96–4.60 (CH–O–CO, m,42H), 4.54 (CH_2_–CH_2_–triazole, NH–CH_2_, d, 4H), 3.89–3.75 (CH_2_–CH_2_–triazole, m, 2H), 3.51 (O–CH_2_–CH_2_–O,s, 546H), 1.45 (CH_2_–CH_2_–CH–CH_2_,m, 247H), 1.20 (CH_2_–CH_2_–CH_2_–CH_3_, m, 246H), 0.80 (CH_3_,s, 133H).

RhB–PEG–b–PDL

^1^HNMR (400 MHz, CDCl_3_) δ (ppm) 7.80 (Triazole–CH, s, 1H), 5.22 (COO–CH_2_–Triazole, s, 2H), 4.89 (CH–O–CO, m, 42H), 4.61–4.51 (CH_2_–CH_2_–triazole, t, 2H), 3.95–3.87 (CH_2_–CH_2_–triazole, t, 2H), 3.66 (O–CH_2_–CH_2_–O, N–CH_2_–CH_3_ s, 500H), 2.45–2.21 (O–CO–CH_2_,m, 85H), 1.78–1.40 (CH_2_–CH_2_–CH–CH_2_, m, 257H), 1.40–1.16 (CH_2_–CH_2_–CH_2_–CH_3_, N–CH_2_–CH_3_m, 270H), 1.00–0.78 (CH_3_, t, 133H).

^1^HNMR (400 MHz, DMSO–d_6_) δ (ppm) 8.24 (Ar–CH, s, 1H), 8.06 (Triazole–CH, s, 1H), 7.66 (Ar–CH,m, 1H), 7.54–7.38 (Ar–CH, m, 1H), 6.54 (Ar–CH,m, 6H), 5.05–4.60 (CH–O–CO, m, 43H), 4.54 (CH_2_–CH_2_–triazole,m, 2H).

#### 2.2.6. Preparation and Characterisation of Blank and Drug-Loaded Mixed Micelles

Two formulations of mixed micelles of synthesised functional copolymers were prepared by decalactone) based the nanoprecipitation method [12]. Briefly, to prepare nonfolate formulation (i.e., PDL micelles), mPEG-b-PDL (10 mg) and RhB-PEG-b-PDL (3 mg) were dissolved in acetone and ethanol mixture (1.5+ 0.5 mL) containing 2 mg of docetaxel (DTX) and added drop-wise into PBS (5.0 mL) under stirring (1000 rpm) and stirred for 6 h at RT. The suspension was then filtered through a 0.22µ syringe filter and used for further analysis. To prepare folate formulation (i.e., PDL-FA micelles), mPEG-b-PDL was replaced with FA-PEG-b-PDL, and blank preparations were prepared in a similar fashion without using the drug.

Micelles’ size and surface charge were measured using a Zetasizer Nano ZS instrument. The concentration of samples used for the analysis contained 70 µg/mL of block copolymer. Samples (in HPLC-grade water) were further imaged using TEM to observe the size and surface morphology. Samples were imaged on TEM copper grids without staining using a JEM 1400-Plus (JEOL Ltd., Tokyo, Japan). Samples were prepared by placing a drop of micelle formulation on the copper grid and dried at RT. The concentration of folic acid (λ_max_—280 nm) and rhodamine B (λ_max_—552 nm) present in the purified micelle solution was measured using UV-Vis spectroscopy. All UV-Vis absorbances were acquired in PBS, and the concentration in micelles was calculated using a prepared calibration curve. The amount of DTX in suspensions was calculated using HPLC. The mobile phase used was water and acetonitrile (45:55), and the column (Gemini-NX 3u C18 110A, 100 × 4.6 mm) temperature was set to 50 °C. The flow rate was set to 1 mL/min, and absorbance was measured at 230 nm. The analysis was performed using a Merck Interface D-7000 Diode Array Detector and samples were run for 10 min to determine the retention time of DTX, which was 4.56 min. The drug concentrations were then calculated using pre-prepared standard calibration curves.

#### 2.2.7. Cell Studies

##### Cell culture and Maintenance

A human triple-negative breast cancer cell line MDA-MB-231 and noncancerous mouse embryonic fibroblast (MEF) cells were cultured with media including Dulbecco’s modified Eagle’s medium (DMEM) supplemented with 10% heat-inactivated foetal bovine serum (FBS), 2 mM L-glutamine, 100 IU/mL penicillin, and 100 µg/mL of Streptomycin (all from Lonza) at 37 °C with 5% CO_2_. Cells were passaged when they reached 80–90% confluency. For folic acid targeting studies, cells were precultured for 24 h with Roswell Park Memorial Institute (RPMI) 1640 medium (Gibco, Cat. No. 27016021) containing no folic acid and supplements mentioned above to induce the overexpression of the folate receptors (FR). After the starvation, the experiments were carried out with culture media.

##### Cytotoxicity of Blank and DTX Loaded Micelles

The cytotoxicity of micelles was evaluated using Alamar Blue cell viability assay (TCI Europe) on MDA-MB-231 cells using reported procedure with minor modifications [13]. Briefly, 100 µL of cell stock suspension, having a concentration of 50,000 cells/mL was seeded into a 96-well plate and incubated for 24 h. Later, PDL micelles were prepared with the concentrations of 0.25, 0.50 and 0.75 mg/mL from stocks in prewarmed (37 °C) growth media. The cell media in 96-well plate was replaced after 24 h with samples and incubated for 48 and 72 h at 37 °C, 5% CO_2_. After incubation, 10 μL of Alamar Blue cell proliferation reagent was added to the wells and incubated for additional 2 h. The fluorescence of samples was then collected according to the manufacturer protocol using 540 nm as excitation and 570–590 nm as emission wavelength. The percentage of cell proliferation was reported compared to untreated cells (100% viability).

A similar procedure was followed to determine the folic acid targeting efficiency of DTX-loaded micelles. Cells were cultured with FA-free media in 96-well-plates for 24 h to induce FR expression. Following day FA-free media was replaced with growth media containing DTX loaded PDL or PDL-FA micelles with the DTX concentrations of 100, 150 and 200nM and cells were treated for 24 h and 48 h to assess the cytotoxicity (n = 4). Corresponding concentrations of free DTX were prepared by dissolving the drug in DMSO with the concentrations of 40.4, 60.6 and 80.8 µg/mL (corresponding to 100, 150 and 250 µM and diluted in the cell media that the final concentration of DMSO would not exceed 0.1% (*v*/*v*) in cell media. Positive control cells were left untreated (cell media only). After incubation, Alamar Blue cell proliferation reagent was utilised for the cell viability assessment.

##### Micelles Internalisation and Endocytosis Route Analysis by Flow Cytometry

MDA-MB-231 cells were cultured in 12-well plates with a concentration of 100,000 cells/mL overnight for attachment. The following day, cell media was replaced with fresh cell media containing 10, 25 and 50 µg/mL of PDL and PDL-FA micelles in triplicates and incubated at 37 °C, 5% CO_2_ for 4 h or 24 h time intervals. Similarly, experiments were also performed at 4 °C for 2 h instead of 37 °C to investigate the energy-dependent uptake of the micelles. After incubation, cells were harvested with trypsinisation, washed and resuspended in PBS. Mean fluorescence intensity of internalised micelles was recorded by using LSRFortessa (BD Sciences, San Diego, CA, USA). The analyser was set to record 20,000 events per sample. Flowing Software (Open source software, Turku Centre for Biotechnology, Finland) was used for data analysis and WinList 9.0 was used for visualisation of the overlay histograms. Acquired data were normalised with the control (untreated cells) and mean fluorescence intensity values were proportioned as fold increase for analysis.

Further, to establish the uptake route of micelles, MDA-MB-231 cells were exposed to various pharmacological inhibitors, including amiloride (75 µM), genistein (400 µM) and phenyl arsine oxide (PAO) (1 µM) and cotreatment of genistein and PAO, in triplicates and their impact on the cellular internalisation of micelles were determined by flow cytometer. Cells were incubated in the 12-well plates at the concentration of 150,000 cells/mL overnight for attachment. Later, cell media was replaced with the inhibitor-containing media and cells were preincubated with the inhibitors for 1h to block the specific endocytosis route. Next, PDL micelles with the 10 µg/mL concentration were added into the inhibitor-containing media and cells were further incubated for 2h. Mean fluorescence intensity of the endocytosed micelles was acquired by flow cytometry, and data were normalised to the uninhibited condition as 100% uptake.

##### Cellular Uptake Determination of PDL and PDL-FA Micelles by Confocal Microscopy

For confocal studies, cells were seeded on sterilised borosilicate glass coverslips and left overnight for attachment with the concentration of 100,000 cells/mL in six-well plates. Attached cells incubated with folic acid starving cell media for 24 h to overexpress the FR. Next, cells were exposed with fresh growth media containing 25 µg/mL of PDL or PDL-FA micelles for 24 h. Cells were washed with PBS after the micelle treatment, and the cell membrane was stained with CellMask™ deep red plasma membrane stain (ThermoFisher Scientific) according to the provided protocol. Briefly, the staining solution was diluted in 1:1000 ratio in 1 mL cell media and cells were incubated in the solution for 10 min at 37 °C. The staining solution was removed, cells were washed and fixed with 4% PFA at RT for 10 min. Membrane stained cells were rinsed with PBS and mounted in VECTASHIELD^®^ MOUNTING MEDIUM with DAPI. Cell images were acquired with Zeiss LSM880 with Airyscan confocal microscopy with the 63× Plan-Apochromat oil objective. Excitation and emission settings were adjusted as following: DAPI λ_Ex_—405 nm, λ_Em_—500–530 nm, Rhodamine λ_Ex_—543 nm, λ_Em_—560–630 nm and deep red cell mask λ_Ex_—633 nm, λ_Em_—660–700 nm. Acquired images were processed by using Fiji ImageJ software and pseudo-coloured with blue (DAPI), red (micelles) and grey (Cell Mask deep red) for visualisation.

### 2.3. Statistical Analysis

Statistical analysis was conducted by employing two-way ANOVA with Tukey’s multiple comparisons test using *p* < 0.05 (95% confidence interval) as a statistical significance threshold unless stated specifically. GraphPad Prism software (version 6) using *n* = 3 was used for all statistical analysis. Statistical significance has been represented as extremely significant (****, *p* < 0.0001), extremely significant (***, *p* = 0.0001–0.001), very significant (**, *p* = 0.001–0.01), significant (*, *p* = 0.01–0.05) and not significant (ns, *p* ≥ 0.05).

## 3. Results and Discussion

### 3.1. Synthesis and Characterisation of Block Copolymers

In this study, block copolymers were synthesised using ring-opening polymerisation (ROP) and click chemistry. In our previous study, we have reported on the generation of undesired homopolymer during the ROP of δ-decalactone and thus, achieving a predefined molecular weight appeared to be a difficult task [13]. Therefore, to retain the identical molecular weight of the hydrophobic block (i.e., PDL) in all synthesised block copolymers, click chemistry was utilised for the synthesis of amphiphilic block copolymers. Copper-catalysed click chemistries are known for efficient reactions at RT and are very sturdy processes to generate regioselective products [23].

**Scheme 1:**
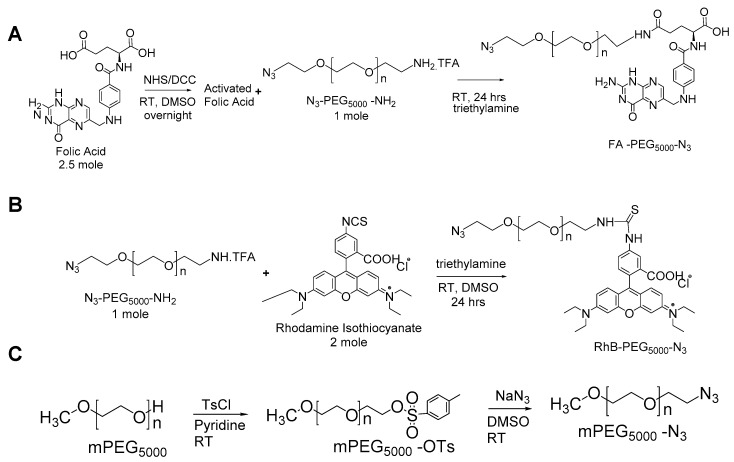
Synthesis scheme of (**A**) Folic acid conjugated poly(ethylene glycol) (FA-PEG-N_3_), (**B**) Rhodamine-B conjugated poly(ethylene glycol) (RhB-PEG-N_3_), and (**C**) Azide terminated poly(ethylene glycol) (mPEG-N_3_), (RT = room temperature, NHS-*N*-hydroxysuccinimide, DCC-*N, N′* dicyclohexylcarbodiimide). Reproduced with permission from [18].

Functionalised block copolymers were synthesised using commercially available N_3_-PEG-NH_2_.TFA, while azide terminated mPEG was prepared in-house to synthesise a nontargeted block copolymer. All block copolymers were synthesised in three steps, i.e., (I) synthesis of azide terminated hydrophilic block (i.e., PEG), (II) synthesis of alkyne terminated hydrophobic block (i.e., PDL) and (III) conjugation of azide and alkyne terminated block by click chemistry. Scheme 1 exemplifies the synthesis methodology utilised to prepare the desired azide terminated PEG.

The conversion of the mPEG-OH into mPEG-azide was expedited by preparing a more reactive mPEG-tosyl intermediate. The emergence of a triplet peak at 4.2 ppm (corresponding to methylene protons next to the tosyl group, Figure S1A, position 4) suggested the attachment of the tosyl group to the PEG [19]. The tosyl group was then replaced with azide by reacting it with sodium azide. The emergence of the triplet at 3.4 ppm (corresponding to the methylene proton next to azide, Figure S1B, position 2), and vanishing of a peak at 4.2 ppm suggested the successful conversion of intermediate into the product (Figure S1B). The NMR analysis indicates that approximately 90% of azide functionality is present in the final product.

Folic acid was conjugated to amine-terminated PEG via an amide bond while rhodamine B was conjugated to the polymer through a thiourea bond [20,21]. Purified PEG conjugates (i.e., FA-PEG-N_3_ and RhB-PEG-N_3_) were characterised by ^1^HNMR and MALDI-TOF MASS spectroscopy. The methylene proton next to the azide group at 3.3 ppm was used as a standard for the relative integration of other peaks (Figure S2, position 7). The proton resonance and peak positions observed in the ^1^HNMR spectrum of FA conjugated PEG were accorded with the values reported in the literature [24,25], suggesting the successful conjugation of folic acid to PEG.

The conjugation of rhodamine B *iso*thiocyanate with PEG was established with ^1^HNMR where the proton resonance of other peaks relating to the methylene proton next to the azide group of PEG (Figure S3, position 3, acquired in CDCl_3_) suggested the successful conjugation of RhB to PEG. The peak positions observed for conjugated RhB were accorded with the previously reported values [26]. FA-PEG-N_3_ and RhB-PEG-N_3_ were further characterised via MASS and SEC. Deviations in the peak shape and position further confirmed the conjugation of FA and RhB to PEG as apparent by MALDI-TOF MASS spectra (Figure S4). The molecular weight (m/z) revealed by the highest peak (100% intensity) detected in MASS spectra was selected to represent the product molecular weight. The molecular weight detected by MASS technique for the conjugates were accorded with the calculated molecular weight.

PEG conjugates of FA and RhB were further analysed on UV-Vis spectroscopy to determine the amount of folic acid and Rhodamine B in the samples. No variation in λ_max_ was observed after the conjugation of FA and RhB with PEG. According to the results, each milligram of FA-PEG-N_3_ contained 81.84 µg of folic acid, whereas 101.65 µg of rhodamine B was present in each milligram of RhB-PEG-N_3_. The percentage conversion based on UV-Vis results suggested 88 and 89.2% of the conjugation of folic acid and rhodamine B to PEG, respectively.

**Scheme 2:**
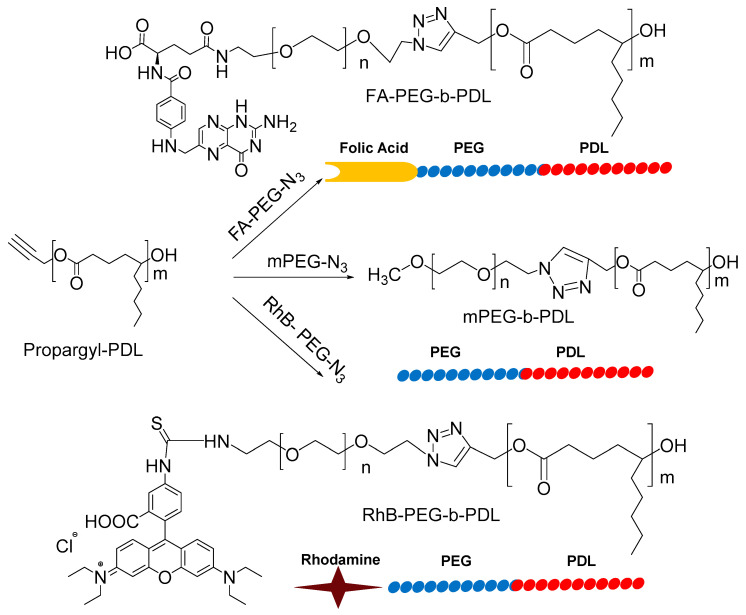
The synthesis scheme of block copolymers of propargyl-PDL via click chemistry. Reproduced with permission from [18].

Alkyne-terminated PDL was synthesised by ROP of δ-decalactone using propargyl alcohol as initiator as per our earlier published procedure [13]. Conjugation of PEG and PDL was achieved via a copper-catalysed click reaction to devise the amphiphilic block copolymers (Scheme 2). Surplus propargyl-PDL was used in the reactions to ensure complete utilisation of azide terminated PEG. A simple solvent extraction was used to separate copper from the product (Figure S5).

The vanishing of the peak in ^1^HNMR at 3.4 ppm (corresponds to CH_2_-N_3_) and the emergence of a new peak at 7.8 (characteristic peak of triazole ring proton) [22], 5.2, 4.5 and 3.9 ppm suggested the efficient conjugation of all azide-terminated PEG to alkyne-terminated PDL (Figures S6–S8). All other peak positions were accorded with the previously reported values [13].

The ^1^HNMR of copolymer FA-PEG-b-PDL and RhB-PEG-b-PDL was also obtained in DMSO to envision the peaks of FA and RhB. Molecular weight via ^1^HNMR was calculated by relating the number of protons at 4.9 ppm (PDL chain, position 3) with respect to the proton resonance of PEG chain at 3.66 or 3.39 ppm (for mPEG) and the proton of the triazole ring at 7.8 ppm (Table 1, Figures S7 and S8). Samples were further analysed via SEC to observe the retention time and an increase in molecular weight (or size) and verify the conjugation of PDL block to the PEG block. However, a shoulder in the mPEG-b-PDL sample and broadened peak in other samples were observed in the SEC traces, which suggested the presence of free PEG. It can be assumed that, due to the presence of approximately 10% of nonazide PEG, the free mPEG and PEG conjugates (i.e., FA-PEG and RhB-PEG) were detected in SEC analysis, which was not reacted with PDL to generate block copolymer (Figure S9, Table 1). The quantity of free PEG and/or PEG conjugates in the final copolymer was anticipated to be ≤10% (based on azide functionalities). Since PEG is a water-soluble polymer, it has been suggested that the presence of PEG_5k_ on the micelles’ surface could reduce the cellular uptake by minimising protein adsorption and, hence, it is highly unlikely that free PEGs would internalise in cells by their own [27,28].

### 3.2. Preparation and Characterisation of Block Copolymer Micelles

**Figure 1:**
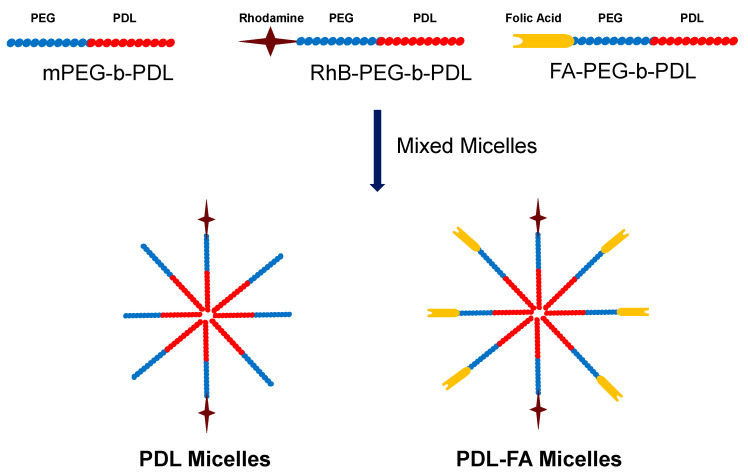
Pictorial presentation of different micelle formulations prepared in this study. PDL micelles were prepared by mixing mPEG-b-PDL (10 mg), and RhB-PEG-b-PDL (3 mg) block copolymers whereas PDL-FA micelles were prepared by mixing FA-PEG-b-PDL (10 mg) and RhB-PEG-b-PDL (3 mg) block copolymers in 5 mL PBS. Reproduced with permission from [18].

Micelles of amphiphilic block copolymers were fabricated via a well-established nanoprecipitation method. PBS (pH 7.4) was used as a solvent for the fabrication of the micelles to ensure the pH-dependent solubility of folic acid in aqueous media. The illustrative presentation of fabricated micelle formulations is shown in Figure 1.

**Figure 2:**
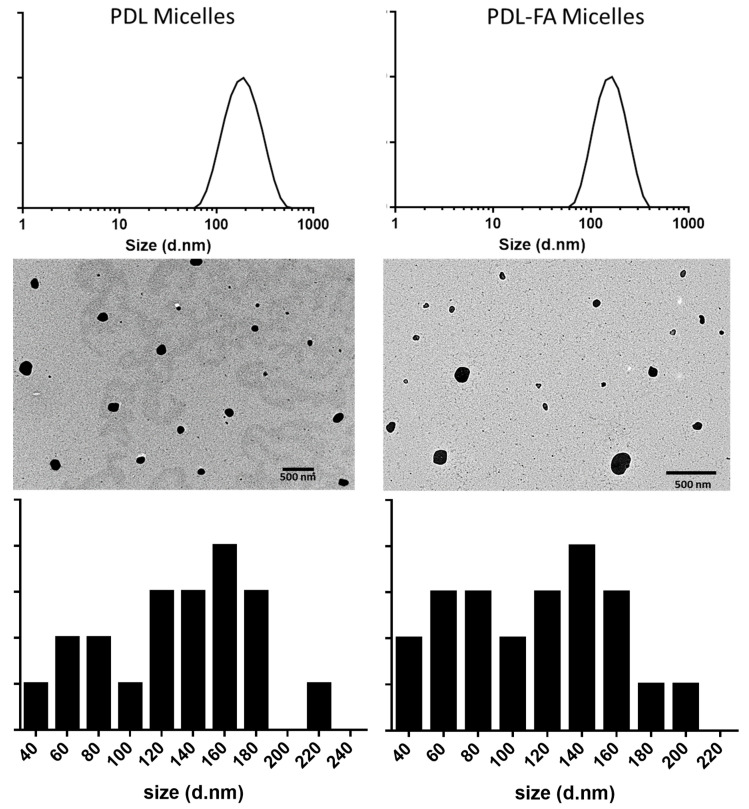
Normalised size distribution by DLS intensity of PDL and PDL-FA micelles (**top**) dispersed in PBS and corresponding TEM image (**middle**, scale bar—500nm) and size distribution histogram from TEM images (**bottom**) (analysed using ImageJ software).

Hydrodynamic size distribution of the fabricated micelles was characterised by DLS, and the statistical analysis (student t-test) of results suggested a nonsignificant difference in Z-average size between both micelle formulations (Figure 2, Table 2). The samples were reanalysed after one week of storage at 4 °C to ascertain the change in size, and the results demonstrated the stability of micelles as aqueous dispersion at least for one week ascertained by absence of aggregation (Figure S10). The zeta potential detected for the PDL micelles in HEPES buffer (pH 7.4) was near to neutral. However, a slight negative charge was detected for PDL-FA micelles compared to PDL micelles, which could be due to the existence of folic acid on the surface of the micelles [29] (Table 2, Figure S11). Both samples were further analysed using TEM to detect the morphology and to verify the size. Images attained from TEM advised that both micelle formulations were roughly spherical in shape with smooth surfaces and in the similar size ranges detected by DLS (Figure 2).

A typical superimposed UV-Vis spectrum of PDL and PDL-FA micelles is presented in Figure S12. In both micelle formulations, the concentration of rhodamine B was found to be 50.2 µg/mL, whereas 123.1 µg/mL of folic acid was available in PDL-FA micelles.

Using a similar technique, DTX-loaded micelles were prepared and purified by filtration to remove the non-entrapped drug. DTX being a hydrophobic molecule, the anticancer potential of DTX is somewhat hindered; thus, several methodologies, including polymeric micelles, have been employed to enhance the aqueous solubility of DTX and eventually, bioavailability. Therefore, DTX was chosen as a model drug in this study to evaluate the efficiency of micelles towards increasing the aqueous solubility of the hydrophobic drug. The concentration of DTX was calculated in formulation using HPLC, and it was observed that PDL micelles contained 61.8 ± 3.6 µg/mL of DTX with percentage entrapment efficiency (%EE) of 30.9 ± 1.8%. In contrast, PDL-FA micelles contained 50.4 ± 4.3 µg (%EE- 25.2 ± 2.2) of DTX per mL of formulation. The results suggested that the PDL and PDL-FA micelles are capable of enhancing the solubility of DTX by 10.8 and 8.8 times, respectively, from represented aqueous solubility [30].

### 3.3. Cytotoxicity and Cellular Uptake of Block Copolymer Micelles in MDA-MB-231 Cells

**Figure 3:**
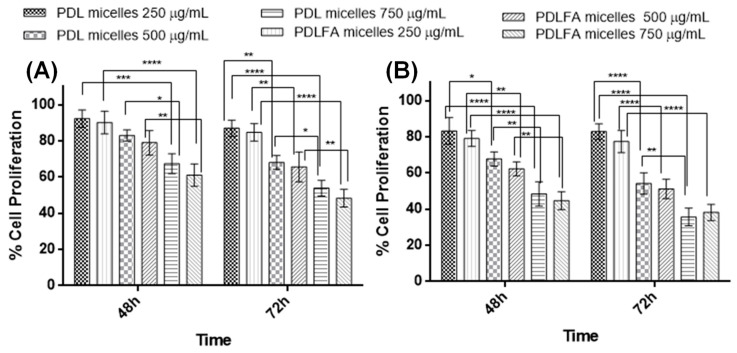
Effect of micelle formulations on (**A**) MDA-MB-231 and (**B**) MEF cells proliferation when incubated for 48 and 72 h with different concentrations of formulation (n = 3). A two-way ANOVA Sidak’s multiple comparisons test was utilised to determine the significant difference. (****, *p* < 0.0001; ***, *p* = 0.0001–0.001; **, *p* = 0.001–0.01; *, *p* = 0.01–0.05).

The cytotoxicity of the novel block copolymer micelles was determined by Alamar Blue cell viability assay to estimate the maximum safe concentration, which can be used for cell studies. Cell viability in MDA-MB-231 cells was established after treatment of PDL and PDL-FA micelles with different concentrations (250, 500, 750 µg/mL) for 48 h and 72 h. As shown in Figure 3, more than 90% of cells were viable when incubated for 48 h with micelles with a concentration of 250 µg/mL, whereas above 80% viability was observed after 72 h incubation. No significant difference was observed in cell proliferation inhibition with 250 µg/mL concentration between both samples and time points. Thus, further cell studies were performed using concentration below 250 µg/mL to avoid any potential cytotoxicity arises from the copolymer micelles. In addition, both formulations were also tested for cytotoxicity on noncancerous MEF cells. The toxicity profiles followed a similar trend as observed for MDA-MB-231 cells but showed slightly higher inhibition in cell proliferation of MEF cells (Figure 3).

Small molecules, such as the drug and dye, are usually taken up by passive transport, whereas nanoparticles are internalised via active transport mechanisms called endocytosis [31]. In order to determine whether the uptake of PDL micelles was an active or passive process, cells were incubated with PDL micelles using different concentrations in parallel at 37 °C and 4 °C (Figure S13). Several enzymes and proteins are known to be sensitive to temperature; thus, active endocytosis processes are inhibited at lowered temperatures. Carrying out the study at 4 °C resulted in robust inhibition of cellular internalisation, showing micelles have been taken up by an energy-dependent process (endocytosis), as suggested in previous studies [32].

**Figure 4:**
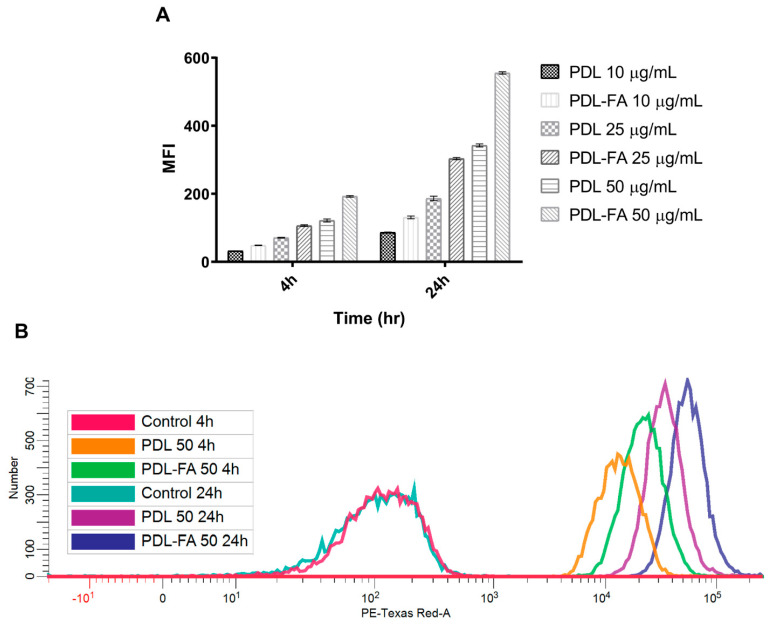
Cellular uptake profile of PDL and PDL-FA micelles after incubation of 4 h and 24 h with MDA-MB-231 cells. Micelles taken up by cells were evaluated by FACS analysis using rhodamine as the fluorescent probe. (**A**) Uptake of PDL micelles was found to be significantly different compared to PDL-FA micelles for each corresponding concentration and time point as analysed by two-way ANOVA, Tukey’s multiple comparisons test (*n* = 3). (**B**) Overlay histograms of PDL and PDL-FA demonstrate the shift upon cellular internalisation.

**Figure 5:**
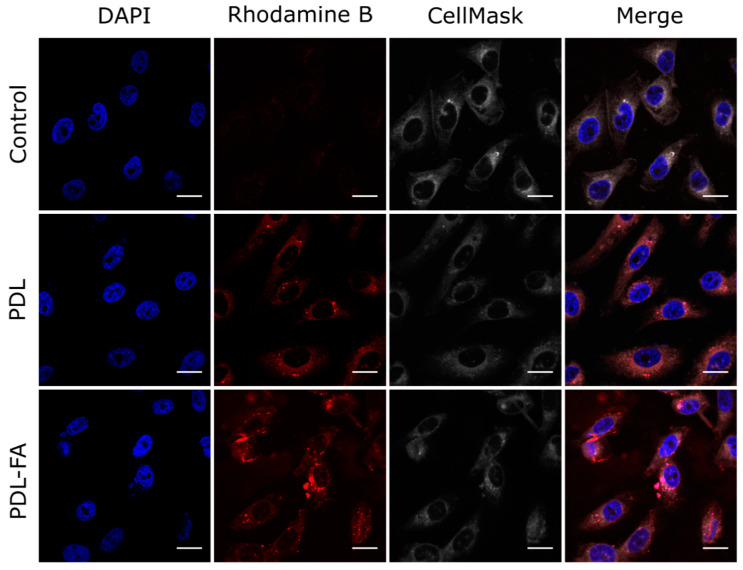
Confocal microscopy images of PDL or PDL-FA micelles internalised in MDA-MB-231 cells after 24 h incubation. Cells were treated 25 µg/mL micelles. Scale bar corresponds to 20 µm. Cell nucleus stained with DAPI (blue), cell membrane stained with CellMask™ Deep Red Plasma Membrane Stain (grey) and red colour depicts rhodamine B conjugated PDL/PDL-FA micelles.

Further, to assess the extent of cellular internalisation of our delivery system and the effect of having folic acid as targeting ligand on cellular uptake efficiency, both micellar formulations were evaluated on the MDA-MB-231 cell line, which is known to have high folate receptor expression [33]. Flow cytometry experiments were performed after inducing the expression of folate receptors of cells by culturing them in folic acid deficient cell media, and later cells were incubated with different concentrations (10, 25, 50 µg/mL) of PDL and PDL-FA micelles in regular growth media for 4h and 24h. The results demonstrated successful cellular internalisation for both FA-tagged and pristine micelles; thus, suggesting the potential of micelles for intracellular delivery. Furthermore, higher cellular uptake of PDL-FA micelles was observed compared with PDL micelles, and the cellular uptake was increased with respect to incubation time and concentration of the sample (Figure 4). Cells exhibited approximately 1.6-fold greater uptake when conjugated with folic acid after 24 h of incubation at 50 µg/mL concentration. Confocal microscopy further confirmed higher cellular uptake for FA-conjugated polymeric micelles (Figure 5). The fluorescence signal in the rhodamine B channel shows that polymeric micelles spread throughout the cytoplasm, and some intracellular aggregation was also observed. Cell Mask is a lipophilic dye used to stain the cell membrane, which was also employed here to indicate the localisation of the micelles. The brighter white spots in the cytoplasm in Figure 5 might implicate the invaginated stained cell membrane as endosomes. The overlapping bright spots in Cell Mask and Rhodamine channel suggest an endosomal localisation of PDL and PDL-FA polymeric micelles in the cells, which would be the expected destination directly after endocytosis. Thus, the observed intracellular aggregates are also most likely due to trapping of micelles inside the endosomal compartments.

Overall, our cellular uptake and microscopy results suggest that cellular uptake of the polymeric micelles in cancer cells can be enhanced via ligand-mediated targeting (FR-mediated endocytosis) using folic acid as targeting ligand. For direct comparison, we have treated a single cell line with targeted and non-targeted micelle formulations rather than utilising FR_+ve_ and FR_-ve_ cell lines; since many other differences between different cell lines will have an impact on the cellular uptake efficiency than solely the level of receptor expression.

**Figure 6:**
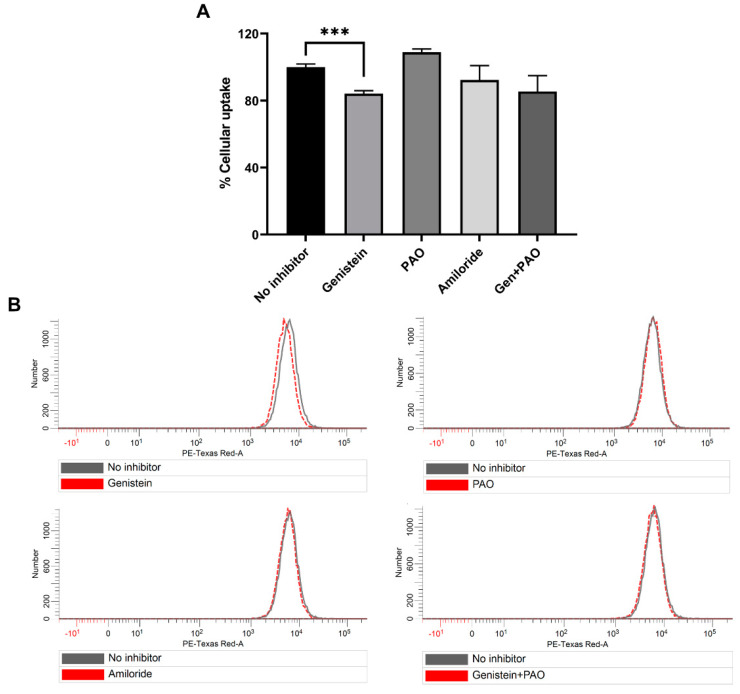
Flow cytometry analysis of various endocytosis inhibitors on uptake of PDL micelles in MDA-MB-231 cells. (**A**) Uptake pathway determination of PDL micelles. Data are expressed as mean ± SEM of three independent experiments. Student’s t-test was applied for statistical analysis compared with respective control without inhibitor for each inhibitor. A significant difference in uptake was observed with the use of genistein which indicates the inhibition of caveolae-mediated endocytosis. (**B**) Histogram overlays of specific inhibitors amiloride, genistein, phenyl arsine oxide (PAO) or combination of genistein+ PAO. The shift in the histogram demonstrates the cellular uptake inhibition by the pharmaceutical compound. (***, *p* = 0.0001–0.001).

Based on the uptake study results, we further investigated the cellular uptake route of our novel PDL micelles, as it is of great importance to determine its entry pathway to establish its biomedical functions, biodistribution and toxicity [34]. Micelles with different size, shape and surface characteristics (charge and hydrophobicity) have different preferred cellular internalisation routes, and endocytosis pathways can be determined by blocking different mechanisms by pharmaceutical inhibitors to investigate the preferred internalisation path. Thus, MDA-MB-231 cells were first exposed to the inhibitors for 1 h to block the various internalisation routes, and later they were co-treated with micelles and various pharmaceutical inhibitors for a further 2 h. The inhibitors included amiloride for macropinocytosis inhibition, phenyl arsine oxide (PAO) for clathrin-mediated endocytosis, and genistein for caveolae-mediated endocytosis. Additionally, genistein and PAO were co-treated to investigate clathrin and caveolae independent endocytosis routes [35]. Inhibitor concentrations were confined to concentrations that did not alter the cell viability (Figure S14). The results showed a significant difference in uptake when inhibited with genistein (Figure 6), thus suggesting caveolae-mediated endocytosis was preferred for PDL micelles. Although it has been suggested that caveolae-mediated endocytosis is less preferred for the particles with size >100 nm due to the size of caveolae, which usually ranges between 50 to 80 nm [34]. However, in an interesting study, micelles with PEG as corona with ~300 nm size followed caveolae-mediated endocytosis for internalisation in cells. This study clearly demonstrates that size is not the only factor responsible for deciding the favourable uptake route for nanocarriers [36]. Earlier, caveolae-mediated endocytosis was also found to be the predominant entry route for 200 nm polystrene nanoparticles [32]. There was no significant alteration in the uptake when cells were co-treated with genistein and PAO, even though there was a distinct decrease when treated with only genistein. This phenomenon could be explained with the survival nature of the cellular system and alteration of the internalisation mechanism, owing to the perturbation of multiple endocytic routes [37].

Caveolae-meditated endocytosis is favourable for nanocarriers as this route is known to bypass lysosomal degradation processes; therefore, vehicles internalised via this route can effectively protect the contents from lysosomal degradation [34]. Additionally, in a study, the uptake of PEGylated liposomal doxorubicin formulation (DOXIL™) was reported via caveolae-mediated endocytosis in MDCK epithelial cancer cells. The diameter of tested DOXIL™ micelles was 85.8 nm with a zeta potential of −2.6 mV at pH 7.4, which is comparable to the PEG-b-PDL micelles [38]. Due to its weakly negative net surface charge, previous studies suggest that negatively charged and neutral nanoparticles induce lysosomal co-localisation [39]. However, our results suggest that the PDL micelles have the capability to bypass the lysosomal degradation, and are therefore holding an excellent promise for intracellular delivery of various biological such as drugs, proteins and genes.

**Figure 7:**
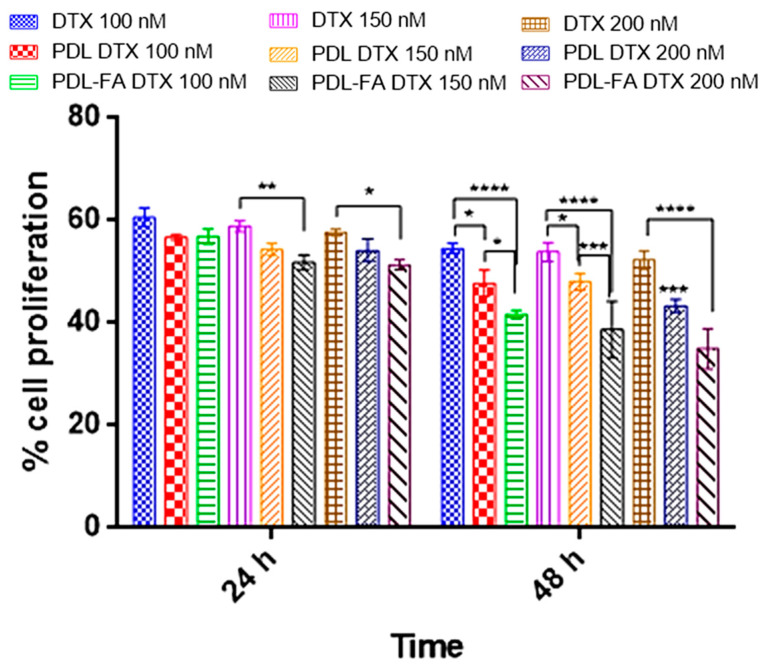
Effect of free DTX and DTX loaded in different micelles formulations on the cell proliferation of MDA-MB-231 when incubated for 24 h and 48 h with varying concentrations of DTX. (****, *p* < 0.0001; ***, *p* = 0.0001–0.001; **, *p* = 0.001–0.01; *, *p* = 0.01–0.05).

To facilitate drug delivery with our novel micelles, and to further establish the advantage of folic-acid-mediated targeting, MDA-MB-231 cells were incubated with DTX-loaded PDL (PDL DTX) and PDL-FA (PDL-FA DTX) micelles and the corresponding concentration of free DTX over 24 h and 48 h. Our results showed no significant difference between the concentrations of 100–200 nM for free DTX regardless of the incubation duration. We speculated that, due to the poor solubility of DTX, an increase in concentration does not have any effect on cell mortality because the saturation solubility has already been reached. Both of the DTX-loaded micelle formulations demonstrated better efficacy towards cancer cell death, particularly after 48h incubation and at higher concentration due to augmentation in the aqueous solubility of DTX (Figure 7). However, despite the increase in aqueous solubility of DTX using micellar formulations, the difference in cytotoxicity after 24 h incubation was trivial. This could be due to the limited release of DTX from micelles in a short duration; owing to the sustained release of the encapsulated drug compared to freely available DTX. In our previous studies, we demonstrate that these kinds of micelles are capable of sustaining the release of hydrophobic drugs such as Amphotericin B, Curcumin and Indomethacin [12,13,14]. However, with a more extended incubation period, more drug was released from micelles and eventually exhibited higher cytotoxicity compared to free DTX. In summary, superior toxicity demonstrated by PDL-FA micelles suggested higher uptake and longer residence time of this formulation imparted via folate receptor-mediated endocytosis.

## 4. Conclusions

The synthesis of amphiphilic diblock copolymers of poly(δ-decalactone) (PEG-b-PDL) containing different functionalities has been attained successfully via ROP and click chemistry. These block copolymers were readily self-assembled into micelles with an approximate size of 150nm. Two mixed micelle formulations using the copolymers were fabricated using a nanoprecipitation method, in which one was actively targeted (PDL-FA micelles), and another was a nontargeted (PDL micelles) formulation. These micelle formulations were tested for cellular uptake efficiency on the MDA-MB-231 cell line. The results suggested higher cellular uptake of PDL-FA micelles, whereas a notable cellular uptake was also observed for PDL micelles. Attempts were made to determine the uptake route of PDL micelles, and it was postulated that the micelles were taken up by cells through caveolae-mediated endocytosis. Micelles were found to improve the intracellular delivery successfully and thus, the cytotoxicity of DTX on the tested cell line. The obtained results suggest that PDL block copolymer micelles are an efficient carrier for intracellular drug delivery and can be readily functionalised for ligand-mediated targeted delivery. The reported methodology could be utilised to generate functional poly(decalactone) copolymers based nanocarriers for the delivery of different hydrophobic drugs with cell surface-specific ligands of interest, thanks to the simplicity of the employed click chemistry. Our future studies will be focused on fabricating micelles with stimuli-sensitive properties to precisely control the drug release before reaching the target site to further highlight the versatility of our drug delivery platform.

## Figures and Tables

**Table 1:** *M_n_* of synthesised copolymers determined by ^1^HNMR and by SEC using chloroform as eluent and polystyrene polymer as reference (Đ- polydispersity).

Sample ID	*M_n_* by ^1^HNMR (KDa)	*M_n_* by SEC (KDa)	Đ by SEC
mPEG-b-PDL	12.2	21.0	1.06
FA-PEG-b-PDL	12.6	23.1	1.15
RhB-PEG-b-PDL	12.7	28.4	1.25

**Table 2:** Z-average size and zeta potential of PDL and PDL-FA micelles. The size of micelles was measured in PBS (10 mM, pH 7.4) whereas zeta potential was measured in HEPES buffer (10 mM, pH 7.4).

Sample	Z-Average Size (d/nm) (± SD)	PDI (± SD)	Zeta Potential (mv) (± SD)
PDL micelles	153 ± 4	0.22 ± 0.02	−2 ± 1
PDL-FA micelles	147± 3	0.14 ± 0.02	−6 ± 1

## Supplementary Materials

The following are available online at https://www.mdpi.com/1999-4923/12/8/726/s1, Figure S1: ^1^HNMR spectra of (A) mPEG-OTs and (B) mPEG-N3 acquired in chloroform-d. Figure S2: ^1^HNMR of FA-PEG-N_3_ in DMSO-d_6_ that contained few drops of D2O. Figure S3: ^1^HNMR of RhB-PEG-N_3_ conjugate acquired in chloroform-d (top) and DMSO-d_6_ (bottom). Figure S4: Overlapped MALDI-TOF MASS spectra of folic acid (FA), rhodamine B (RhB) conjugated PEG and non-conjugated PEG. Figure S5: Picture of separated copper at bottom of eppendorf after centrifugation at 15000 rpm for 2 min. Figure S6: (A) ^1^HNMR spectra of mPEG-b-PDL synthesised by click reaction and (B) overlapped ^1^HNMR spectra of mPEG-N_3_, mPEG-b-PDL, FA-PEG-N_3_ and FA-PEG-b-PDL. Figure S7: ^1^HNMR spectra of FA-PEG-b-PDL acquired in chloroform-d and DMSO-d_6_. Figure S8: ^1^HNMR spectra of RhB-PEG-b-PDL acquired in chloroform-d and DMSO-d6. Figure S9: Overlapped SEC traces of various PEG-b-PDL copolymers. Figure S10: Normalized size distribution by intensity of PDL and PDL-FA micelles dispersed in PBS for day 1 and day 7 to check the stability in aqueous dispersion. Figure S11: Zeta potential distribution of (A) PDL and (B) PDL-FA micelles in HEPES buffer (10 mM, pH-7.4). Figure S12: UV-Visible spectra of PDL and PDL-FA micelles acquired using PBS as solvent. Figure S13: PDL uptake in MDA-MB-231 cells at 4 °C and 37 °C to determine whether micelles uptake was an active or passive process, investigated by flow cytometry (n = 3). Figure S14 Determination of the cell viability by incubating endocytosis inhibitors phenylarsine oxide (PAO), amiloride (Amil), genistein (Gen) for 3 h in MDA-MB-231 cells.
