# Supplementary material for: Assessment of Intracellular Delivery Potential of Novel Sustainable Poly(δ-decalactone)-Based Micelles
Source: Pharmaceutics. 2020 Aug 2;12(8):726. doi: 10.3390/pharmaceutics12080726 (PMC7465297; doi:10.3390/pharmaceutics12080726)
Supplement: Supplementary file 1 [file pharmaceutics-12-00726-s001.pdf]

pharmaceutics

Supplementary Materials: Assessment of Intracellular Delivery Potential of Novel Sustainable Poly(δ-decalactone)-Based Micelles

Kuldeep Kumar Bansal, Ezgi Özliseli, Gaurav Kumar Saraogi and Jessica M. Rosenholm

Table of Content:

Figure S1 ^{1}HNMR spectra of (A) mPEG-OTs and (B) mPEG-N_3 acquired in chloroform-d. Inset in figure B showing the appearance of triplet peak at 3.4 ppm. 2

Figure S2 ^{1}HNMR of FA-PEG-N_3 in DMSO-d_6 that contained few drops of D_2O. The water peak generally observed at 3.3 ppm in DMSO, which was shifted to 3.7 due to the presence of D_2O.......... 3

Figure S3 ^{1}HNMR of RhB-PEG-N_3 conjugate acquired in chloroform-d (top) and DMSO-d_6 (bottom). ^{1}HNMR spectra in DMSO-d_6 was acquired to visualize the peak of rhodamine B. 4

Figure S4 Overlapped MALDI-TOF MASS spectra of folic acid (FA), rhodamine B (RhB) conjugated PEG and non-conjugated PEG. 5

Figure S5 Picture of separated copper at bottom of eppendorf after centrifugation at 15,000 rpm for 2 min. Copolymer (FA-PEG-b-PDL) solution was made in chloroform at a concentration of 100 mg/mL. 5

Figure S6 (A) ^{1}HNMR spectra of mPEG-b-PDL synthesised by click reaction and (B) overlapped ^{1}HNMR spectra of mPEG-N_3, mPEG-b-PDL, FA-PEG-N_3 and FA-PEG-b-PDL. Disappearance of peak at 3.42 ppm and appearance of peak at 3.90 ppm suggesting the conversion of azide in to triazole... 6

Figure S7 ^{1}HNMR spectra of FA-PEG-b-PDL acquired in chloroform-d and DMSO-d_6. Due to poor solubility of folic acid in chloroform-d, a ^{1}HNMR spectra of FA-PEG-b-PDL was also acquired in DMSO-d_6 to visualise the folic acid peaks between 6.5–9.0 ppm. 7

Figure S8 ^{1}HNMR spectra of RhB-PEG-b-PDL acquired in chloroform-d and DMSO-d_6. Due to poor solubility of rhodamine B in chloroform-d, ^{1}HNMR spectra of RhB-PEG-b-PDL was also acquired in DMSO-d_6 to visualise the rhodamine peaks between 6.0–8.5 ppm 8

Figure S9 Overlapped SEC traces of various PEG-b-PDL copolymers. Chloroform was used as mobile phase and M_n was calculated against polystyrene polymer as reference. 9

Figure S10 Normalized size distribution by intensity of PDL and PDL-FA micelles dispersed in PBS for day 1 and day 7 to check the stability in aqueous dispersion. 9

Figure S11 Zeta potential distribution of (A) PDL and (B) PDL-FA micelles in HEPES buffer (10 mM, pH-7.4). The concentration of samples used for analysis was 70 μg/mL. 10

Figure S12 UV-Visible spectra of PDL and PDL-FA micelles acquired using PBS as solvent. 10

Figure S13 PDL uptake in MDA-MB-231 cells at 4 °C and 37 °C to determine whether micelles uptake was an active or passive process, investigated by flow cytometry (*n* = 3). 11

Figure S14 Determination of the cell viability by incubating endocytosis inhibitors phenylarsine oxide (PAO), amiloride (Amil), genistein (Gen) for 3 h in MDA-MB-231 cells..........11

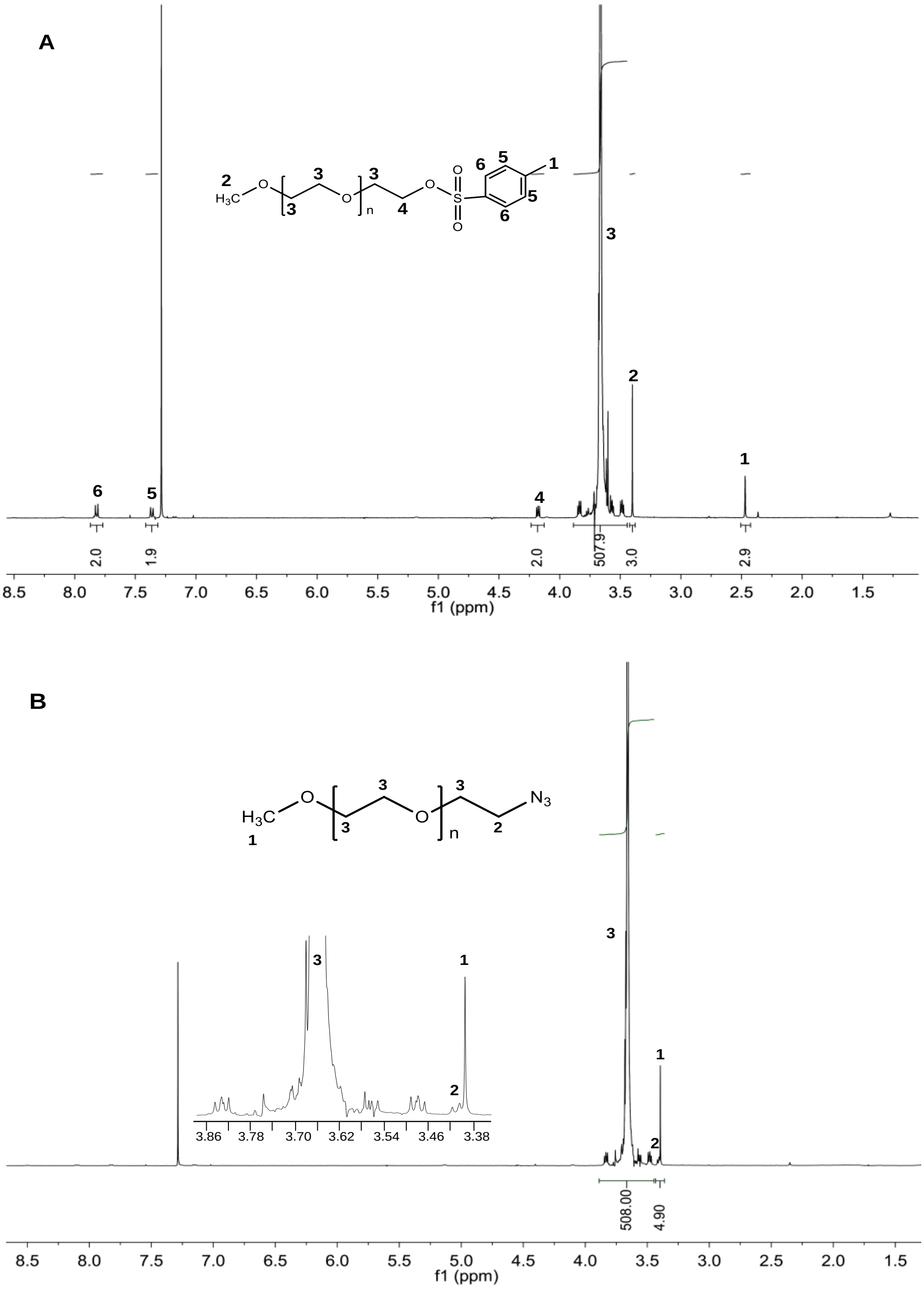

Figure S1. ^{1}HNMR spectra of (**A**) mPEG-OTs and (**B**) mPEG-N_3 acquired in chloroform-d. Inset in figure B showing the appearance of triplet peak at 3.4 ppm.

The appearance of a triplet peak at 4.2 ppm (corresponding to methylene protons next to the tosyl group, Figure S1A, position 4) suggested the attachment of the tosyl group to the PEG. Later, the tosyl group was replaced with azide by reacting with sodium azide. The appearance of the triplet at 3.4 ppm (corresponding to the methylene proton next to azide, Figure S1B, position 2), and disappearance of peak at 4.2 ppm suggested the successful conversion of intermediate into the product (Figure S1B).

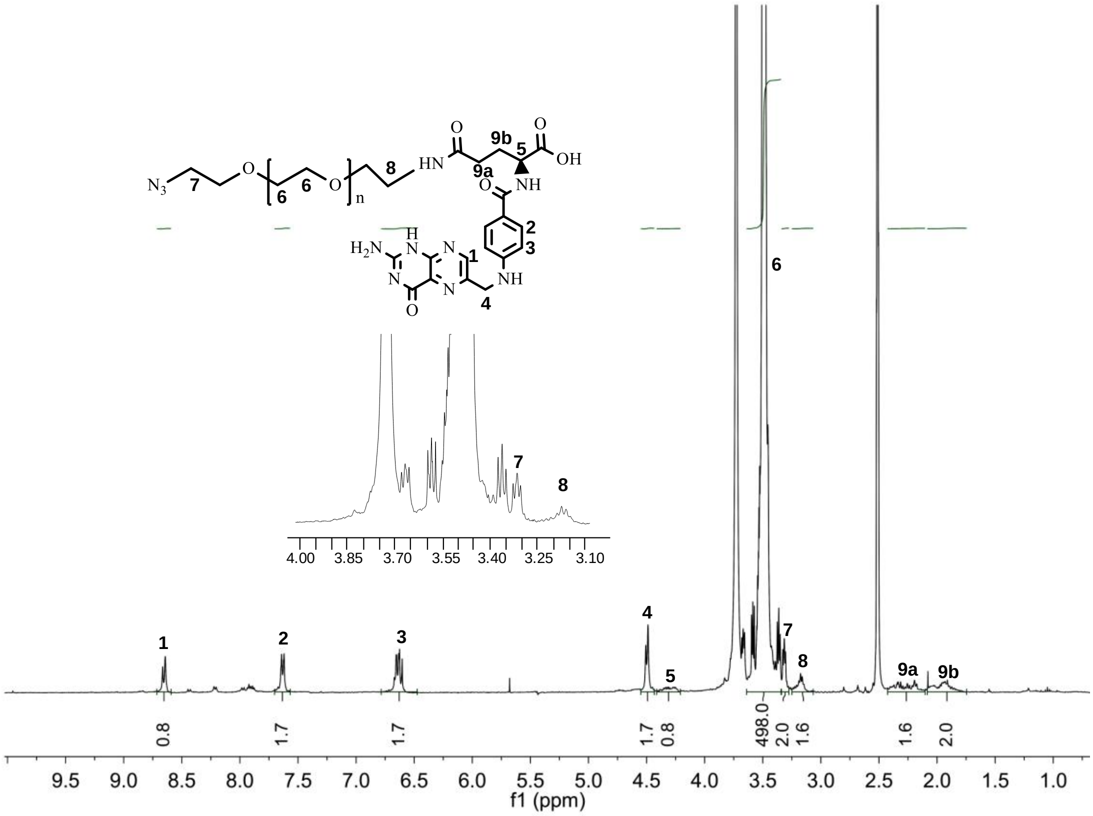

Figure S2. ^{1}HNMR of FA-PEG-N_3 in DMSO-d_6 that contained few drops of D_2O. The water peak generally observed at 3.3 ppm in DMSO, which was shifted to 3.7 due to the presence of D_2O.

The methylene proton next to the azide group at 3.3 ppm was used as a standard for the comparative integration of other peaks.

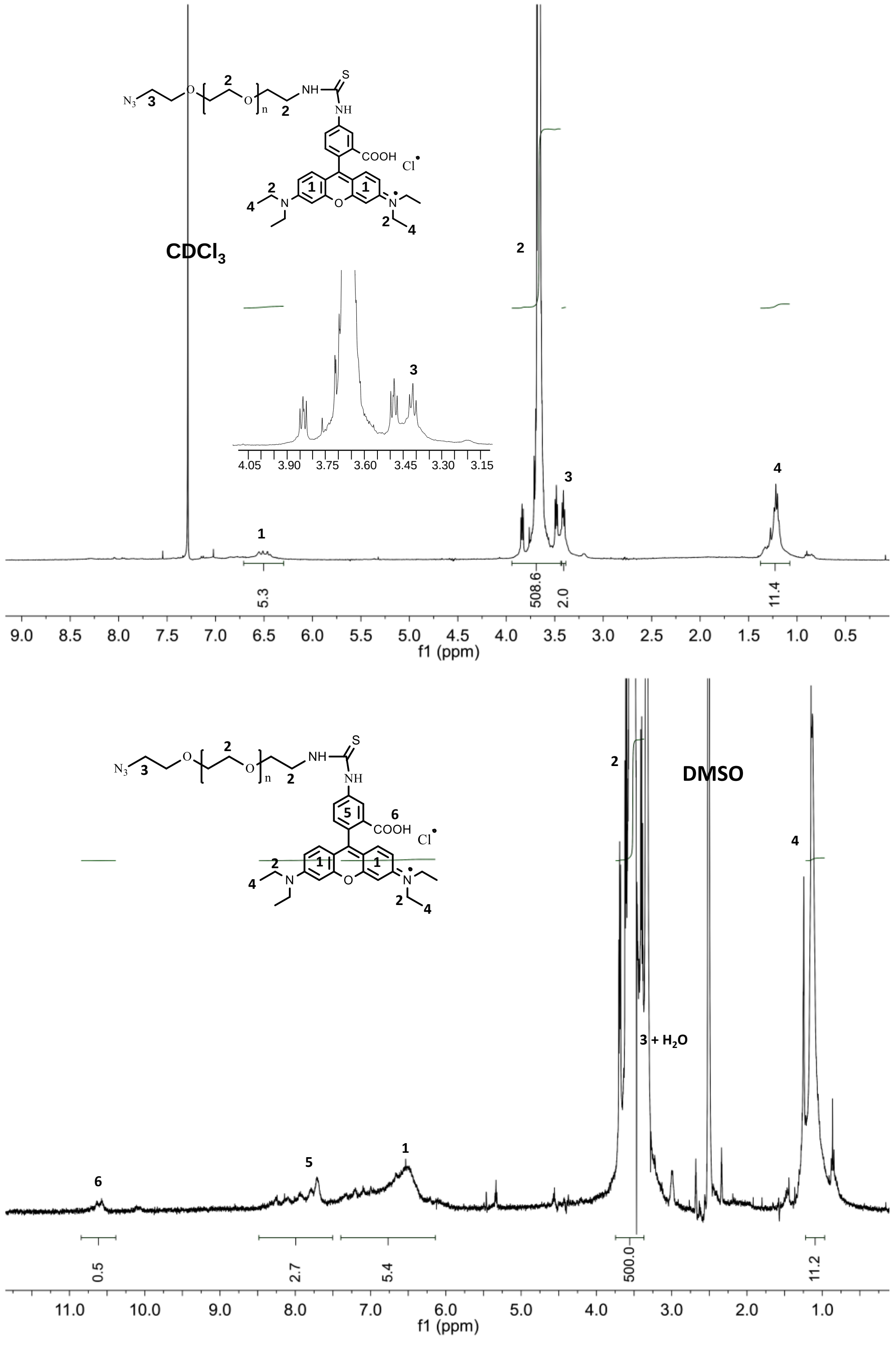

Figure S3. ^{1}HNMR of RhB-PEG-N_3 conjugate acquired in chloroform-d (top) and DMSO-d_6 (bottom). ^{1}HNMR spectra in DMSO-d_6 was acquired to visualize the peak of rhodamine B.

The proton resonance of other peaks concerning the methylene proton next to the azide group of PEG (Figure S3, position 3, acquired in $CDCl_3$) suggested the successful conjugation of RhB to PEG.

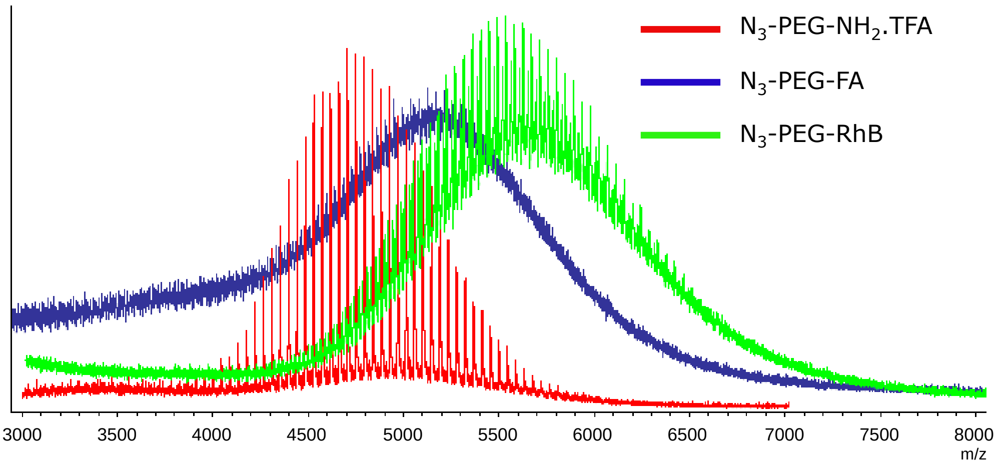

Figure S4. Overlapped MALDI-TOF MASS spectra of folic acid (FA), rhodamine B (RhB) conjugated PEG and non-conjugated PEG.

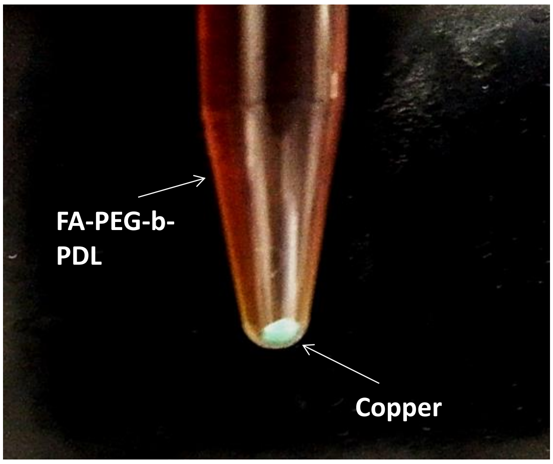

Figure S5. Picture of separated copper at bottom of eppendorf after centrifugation at 15,000 rpm for 2 min. Copolymer (FA-PEG-b-PDL) solution was made in chloroform at a concentration of 100 mg/mL.

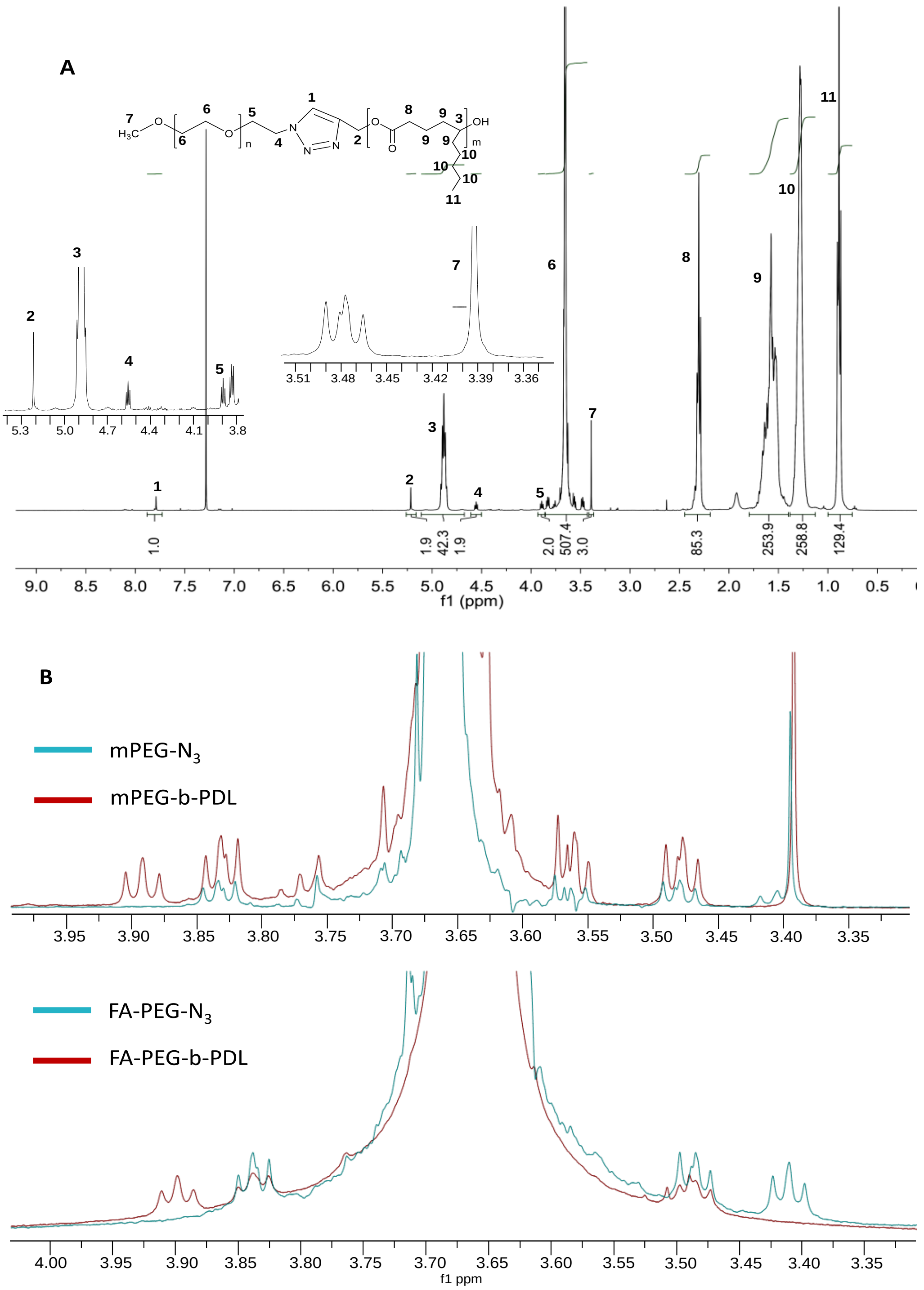

Figure S6. (A) ^{1}HNMR spectra of mPEG-b-PDL synthesised by click reaction and **(B)** overlapped ^{1}HNMR spectra of mPEG-N_3, mPEG-b-PDL, FA-PEG-N_3 and FA-PEG-b-PDL. Disappearance of peak at 3.42 ppm and appearance of peak at 3.90 ppm suggesting the conversion of azide in to triazole.

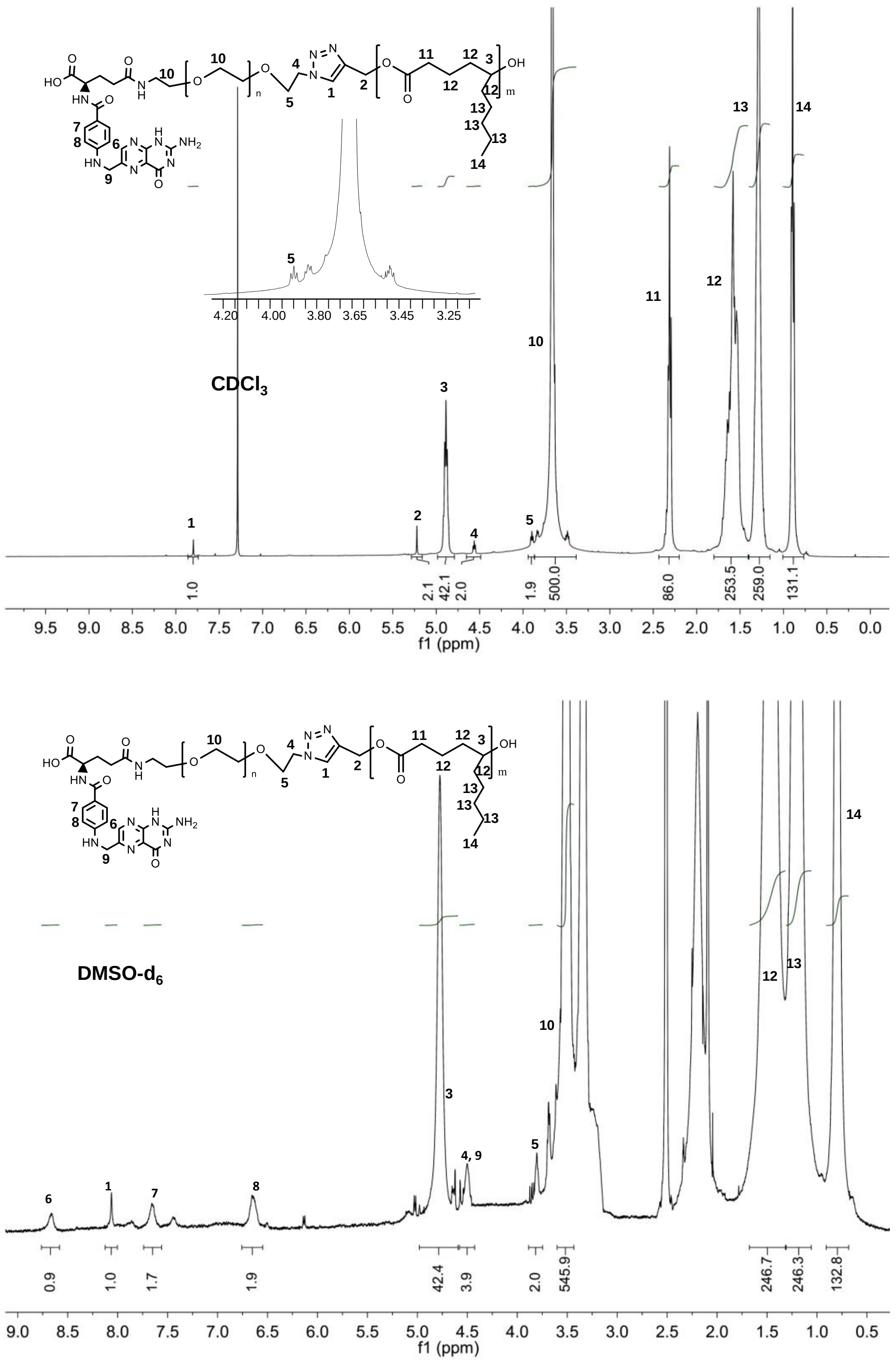

Figure S7. ^{1}HNMR spectra of FA-PEG-b-PDL acquired in chloroform-d and DMSO-d$_6$. Due to poor solubility of folic acid in chloroform-d, a ^{1}HNMR spectra of FA-PEG-b-PDL was also acquired in DMSO-d$_6$ to visualise the folic acid peaks between 6.5–9.0 ppm.

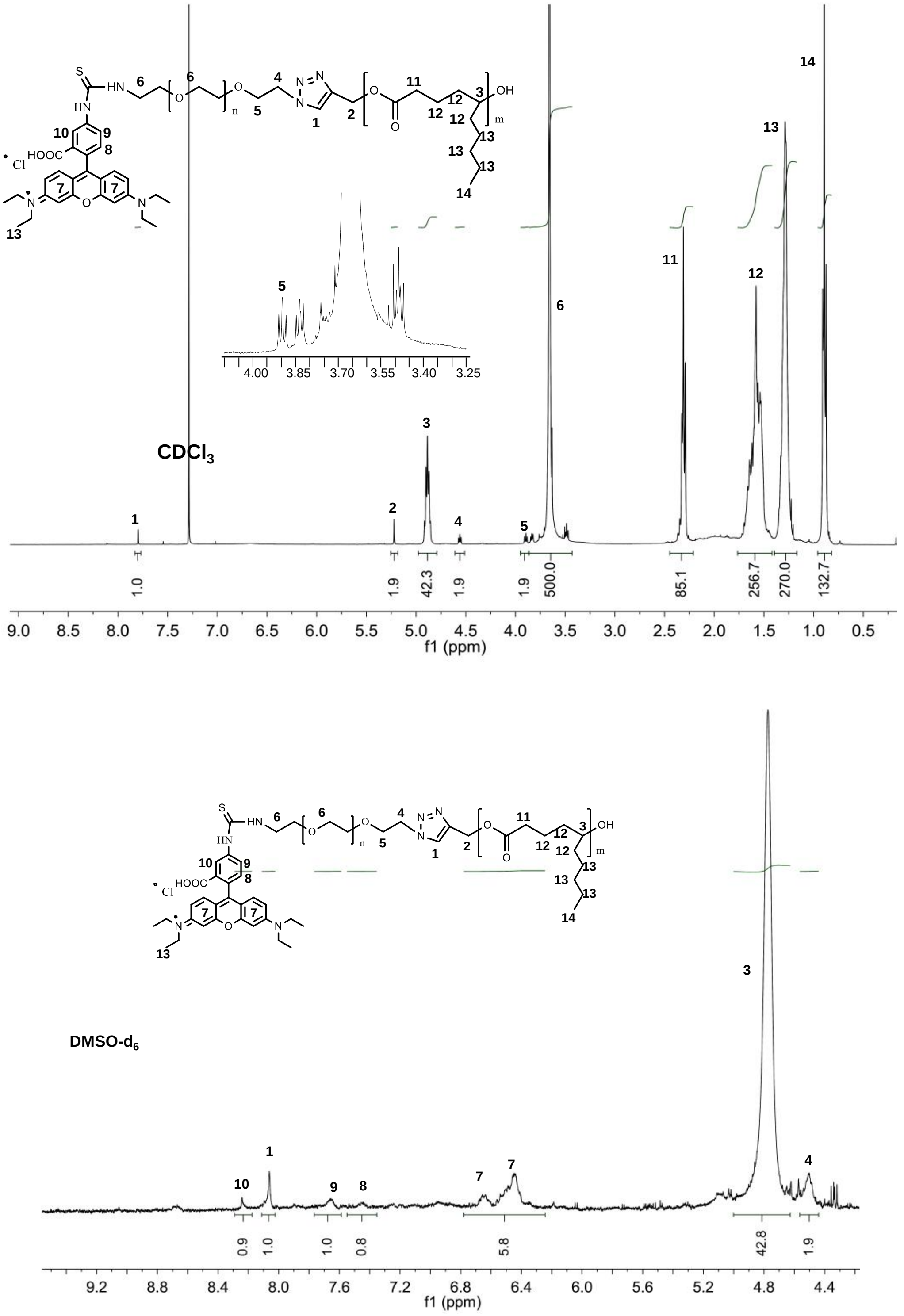

Figure S8. ^{1}HNMR spectra of RhB-PEG-b-PDL acquired in chloroform-d and DMSO-d$_6$. Due to poor solubility of rhodamine B in chloroform-d, ^{1}HNMR spectra of RhB-PEG-b-PDL was also acquired in DMSO-d$_6$ to visualise the rhodamine peaks between 6.0–8.5 ppm.

The disappearance of the peak in ^{1}HNMR at 3.4 ppm (corresponds to C<u>H</u>$_2$-N$_3$) and appearance of a new peak at 7.8 (characteristic peak of triazole ring proton) 5.2, 4.5 and 3.9 ppm suggested the successful conjugation of all azide terminated PEG to alkyne terminated PDL. The ^{1}HNMR of copolymer FA-PEG-b-PDL and RhB-PEG-b-PDL were also acquired in DMSO to visualise the peaks of FA and RhB. Molecular weight via ^{1}HNMR was calculated by comparing the number of protons

at 4.9 ppm (PDL chain, position 3) with respect to the proton resonance of PEG chain at 3.66 or 3.39 ppm (for mPEG) and the proton of the triazole ring at 7.8 ppm (Figure S6–S8)

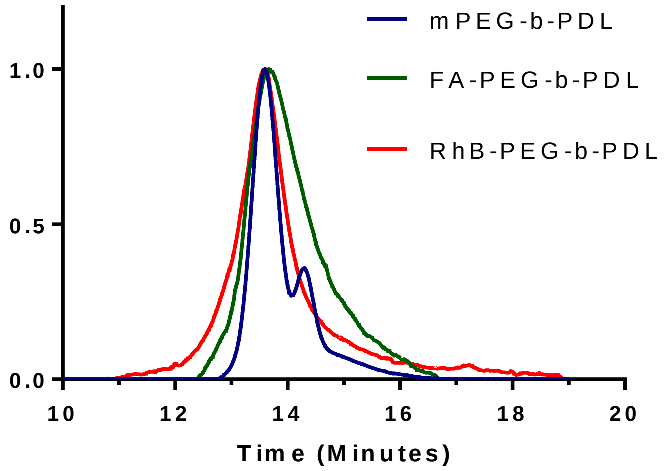

Figure S9. Overlapped SEC traces of various PEG-b-PDL copolymers. Chloroform was used as mobile phase and M_n was calculated against polystyrene polymer as reference.

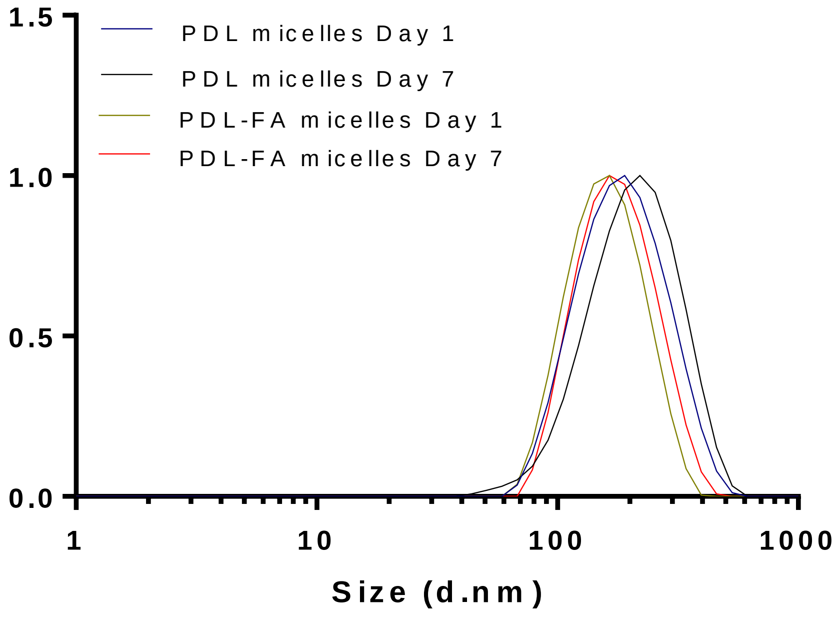

Figure S10. Normalized size distribution by intensity of PDL and PDL-FA micelles dispersed in PBS for day 1 and day 7 to check the stability in aqueous dispersion.

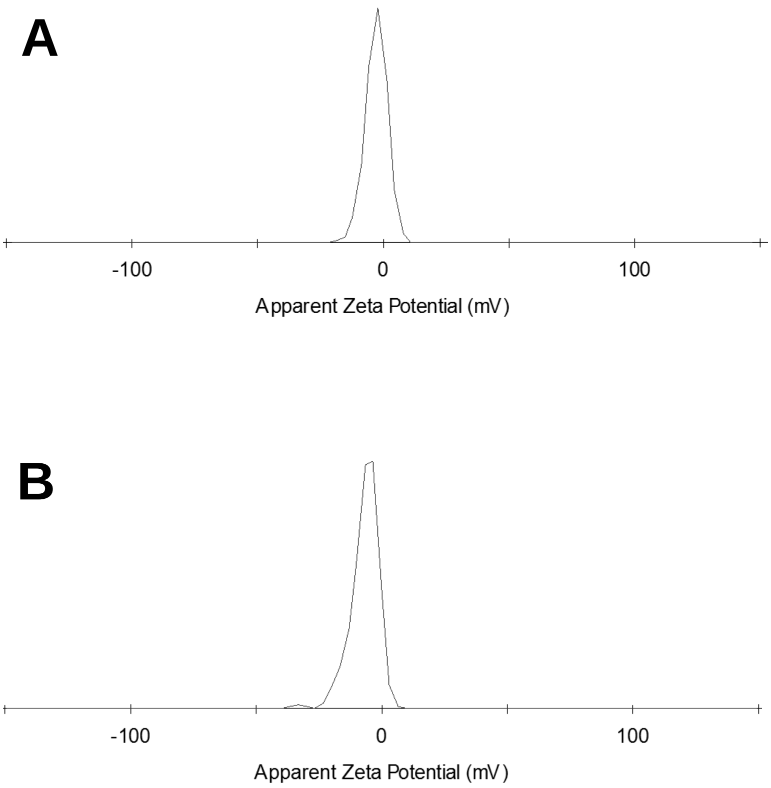

Figure S11. Zeta potential distribution of (**A**) PDL and (**B**) PDL-FA micelles in HEPES buffer (10mM, pH-7.4). The concentration of samples used for analysis was 70 µg/mL.

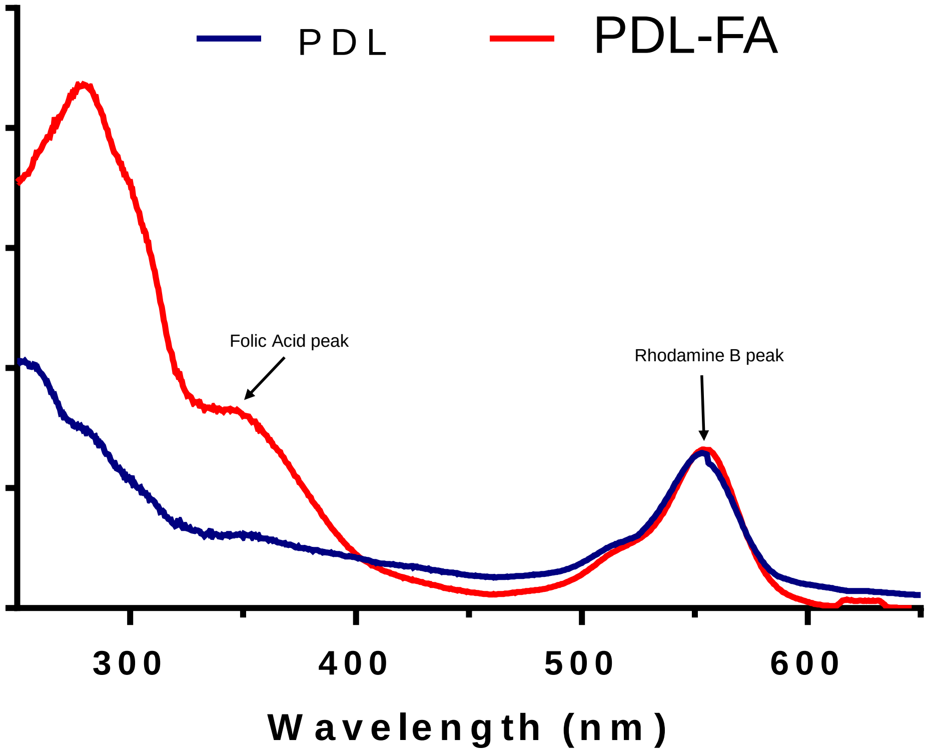

Figure S12. UV-Visible spectra of PDL and PDL-FA micelles acquired using PBS as solvent.

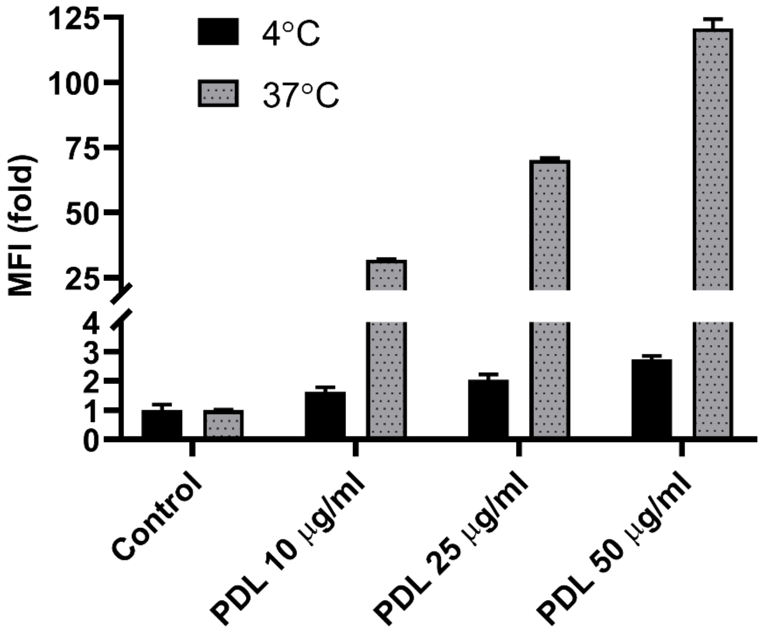

Figure S13. PDL uptake in MDA-MB-231 cells at 4 °C and 37 °C to determine whether micelles uptake was an active or passive process, investigated by flow cytometry (*n* = 3).

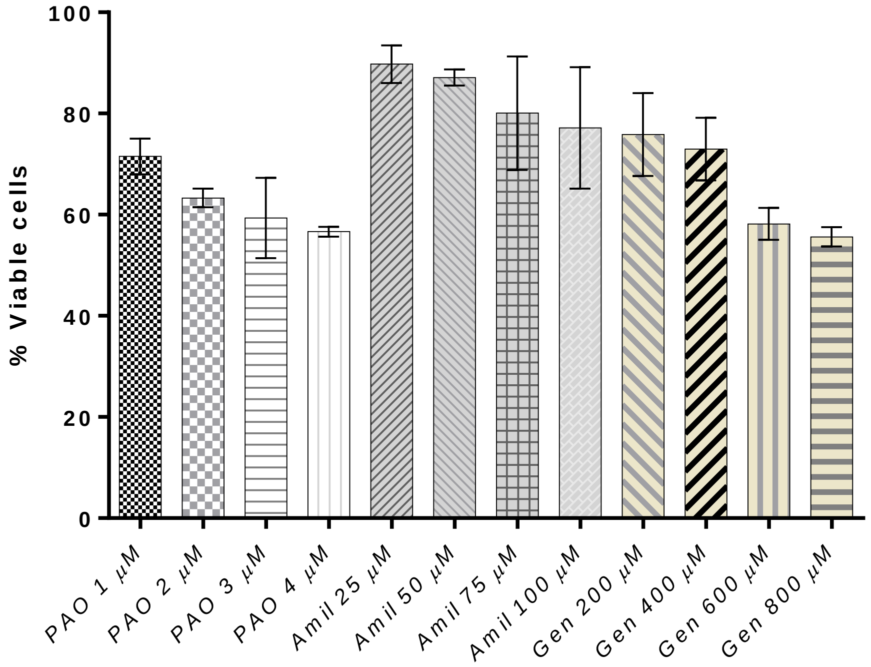

Figure S14. Determination of the cell viability by incubating endocytosis inhibitors phenylarsine oxide (PAO), amiloride (Amil), genistein (Gen) for 3 h in MDA-MB-231 cells.
